# The impacts of collaboration between local health care and non-health care organizations and factors shaping how they work: a systematic review of reviews

**DOI:** 10.1186/s12889-021-10630-1

**Published:** 2021-04-19

**Authors:** Hugh Alderwick, Andrew Hutchings, Adam Briggs, Nicholas Mays

**Affiliations:** 1 Health Foundation, 8 Salisbury Square, London, EC4Y 8AP UK; 2 London School of Hygiene and Tropical Medicine, 15-17 Tavistock Place, London, WC1H 9SH UK; 3 University of Warwick, Coventry, CV4 7AL UK

**Keywords:** Inter-organizational collaboration, Multisector partnerships, Systematic review, Health policy

## Abstract

**Background:**

Policymakers in many countries promote collaboration between health care organizations and other sectors as a route to improving population health. Local collaborations have been developed for decades. Yet little is known about the impact of cross-sector collaboration on health and health equity.

**Methods:**

We carried out a systematic review of reviews to synthesize evidence on the health impacts of collaboration between local health care and non-health care organizations, and to understand the factors affecting how these partnerships functioned. We searched four databases and included 36 studies (reviews) in our review. We extracted data from these studies and used Nvivo 12 to help categorize the data. We assessed risk of bias in the studies using standardized tools. We used a narrative approach to synthesizing and reporting the data.

**Results:**

The 36 studies we reviewed included evidence on varying forms of collaboration in diverse contexts. Some studies included data on collaborations with broad population health goals, such as preventing disease and reducing health inequalities. Others focused on collaborations with a narrower focus, such as better integration between health care and social services. Overall, there is little convincing evidence to suggest that collaboration between local health care and non-health care organizations improves health outcomes. Evidence of impact on health services is mixed. And evidence of impact on resource use and spending are limited and mixed. Despite this, many studies report on factors associated with better or worse collaboration. We grouped these into five domains: motivation and purpose, relationships and cultures, resources and capabilities, governance and leadership, and external factors. But data linking factors in these domains to collaboration outcomes is sparse.

**Conclusions:**

In theory, collaboration between local health care and non-health care organizations might contribute to better population health. But we know little about which kinds of collaborations work, for whom, and in what contexts. The benefits of collaboration may be hard to deliver, hard to measure, and overestimated by policymakers. Ultimately, local collaborations should be understood within their macro-level political and economic context, and as one component within a wider system of factors and interventions interacting to shape population health.

**Supplementary Information:**

The online version contains supplementary material available at 10.1186/s12889-021-10630-1.

## Background

Collaboration between health care, social services, and other sectors is increasingly seen as a route to improving health and health equity [1–5]. The reasons for this are not hard to find. Population health is influenced by a broad range of factors—including structural social and economic conditions, public policies on education, social security, health care, and other areas, living and working environments, and more [6, 7]. While access to health care is an important part of this picture, wider non-medical factors, such as education and income, play a major role in shaping health and its distribution [8–15]. These factors, in turn, are influenced by the activities of multiple organizations and groups, such as national and local governments, social services agencies, schools, and employers. Cross-sector partnerships have been proposed as a way to coordinate these activities to improve people’s health.

Collaboration between sectors to improve health is nothing new. Health in all policies approaches, for example—where health impacts are considered in policy processes across government—have been developed by governments in Europe, Canada, and elsewhere [16, 17]. International collaborations have long been used to help address public health challenges, such as tobacco control and tackling poverty [18, 19]. And various local and regional partnerships to improve health have been established in diverse contexts [20–22]. This includes joint working between health care and social services [23–25], wider public service partnerships [4, 26, 27], and community coalitions of diverse stakeholders [28–30]—sometimes covering states or counties, and sometimes targeting smaller populations in cities or neighbourhoods. These collaborations can be voluntary, mandated, or developed in response to national policy.

Despite their long history, little is known about the impact of cross-sector partnerships between local agencies on health outcomes [4, 26, 31]. Multiple studies and reports have identified potential characteristics of effective partnership working, such as trust and shared objectives between organizations and their leaders [23, 24, 32–34]. But evidence that these partnerships actually achieve their stated objectives—improvements in health or reductions in health inequalities—is hard to find. Many partnerships end up being costly, hard to manage, and struggle to navigate the various cultural, organizational, and accountability issues they face [35]. When subject to closer inspection, even the most mature partnerships can appear less robust and ready to transform their community’s health than their reputations might suggest [36].

Lack of evidence on effectiveness of local partnership working has not deterred policymakers from promoting it. Cross-sector collaboration is currently in vogue among policymakers in the US, UK, and elsewhere—often linked to a growing interest in the health care system’s role in addressing the social determinants of health [7]. In England, for example, sustainability and transformation partnerships have been established between health care organizations, social services organizations, and public health in 42 areas of England (covering populations of around one to three million), with the aim of improving health and making better use of local resources [37]. These partnerships build on a long history of efforts to better coordinate health and social services to improve health in England, including Health Action Zones, Local Strategic Partnerships, Integrated Care and Support Pioneers, and more [35, 38]. In the US, collaborations between health care and non-health agencies are being encouraged through federal programs [39], state initiatives [40], and alternative payment models [41]. Partnership between local agencies has also been a core part of the COVID-19 response—and collaboration is likely to remain a policy mechanism of choice as health systems recover from the pandemic.

The logic behind these policy initiatives varies. Under the right conditions, theory suggests that organizations may achieve better results by combining their skills and capabilities [33, 42, 43]. From a resource dependence perspective [44, 45], partnerships offer organizations opportunities to access new skills, manage interdependencies, and share risks. Partnerships may also help improve efficiency by reducing transaction costs [42, 46–48]. At the same time, partnerships bring their own risks, such as coordination problems, conflicting goals, and loss of power. As organizations collaborate, competing institutional rules and norms may come into conflict [49, 50]. And despite the best efforts of local organizations and the individuals within them, local partnerships are shaped by the broader political economy in which they operate [51].

So how do we make sense of existing evidence to inform today’s policies on collaboration? The literature on organizational collaboration and health is vast and varied, including several reviews of different kinds of partnership working. Yet there is no up-to-date synthesis of the evidence on the impacts of partnerships between local health care and non-health care organizations, and the factors shaping their success. There is also no overarching review of reviews on the mix of evidence related to organizational collaboration and health. We systematically review evidence on the impact of collaboration between local health care and non-health care organizations, as well as the factors shaping partnership functioning.

## Methods

### Design

We carried out a systematic review of reviews to synthesize qualitative and quantitative evidence on the health impacts of collaboration between local health care and non-health care organizations, as well as to understand the factors affecting the functioning of organizational partnerships focused on improving health. Unlike most umbrella reviews [52], which review systematic reviews only, we reviewed systematic and other reviews (such as scoping reviews) of relevant literature. This is because we wanted to identify evidence on how and why partnerships may succeed or fail, not just data on effectiveness. The search strategy was developed with a health services research information specialist and reviewed using Peer-Review for Electronic Search Strategies guidance [53], with feedback incorporated into the strategy. The protocol for the systematic review was not registered.

### Literature search

We conducted searches in Medline, Embase, Web of Science Social Sciences Citation Index, and Health Management Information Consortium for relevant studies (reviews) in English published between January 1999 and December 2019. We screened reference lists of relevant papers and contacted experts to identify potential further studies for inclusion. The search strategy was tested in Medline to ensure that key ‘tracer papers’ were found in our searches [54]. Additional File 1 outlines our search strategy in Medline. For the purposes of the search, we defined collaboration as activities between distinct organizations working together to achieve health goals, including through formal and informal partnership arrangements. This relatively inclusive definition of collaboration was adopted, in part, to reflect the body of literature that we sought to review, where organizational collaboration is often broadly defined and multiple forms of collaboration are typically studied together (see Additional File, Table S1).

### Study selection

Inclusion criteria were developed to identify relevant studies (see Table 1). These focused on identifying reviews of empirical evidence related to collaborations between two or more distinct organizations, collaborations involving health care and non-health organizations at a sub-national level, and collaborations focused on improving health or reducing inequalities. A key aim of the review was to understand factors affecting the success of organizational partnerships, not just their health impacts. We therefore included studies reporting data on partnership mechanisms and processes affecting the success of relevant organizational collaborations, even if they did not report the impact of these collaborations on outcomes.

**Table 1:** Inclusion and exclusion criteria for the systematic review

Include if the study:	
- Focuses on collaboration between two or more distinct organizations that aims to improve health-related outcomes. Health-related outcomes includes improvements in services, such as care quality, as well as impacts on health outcomes and inequalities.	
- Focuses on collaborations at a local level—meaning that the collaborations operate primarily at a sub-national level, such as a state, region, county, or neighbourhood.	
- Focuses on collaborations with at least one health care organization (eg a hospital or primary care practice), and at least one non-health care organization (eg local government, housing, social services, or transportation agencies).^a^	
- Is a systematic or other type of scholarly review of empirical data on collaboration outcomes or processes and mechanisms that may affect collaboration outcomes.	
Exclude if the study:	
- Focuses on collaboration between professional groups within single organizations, or within merged organizations (even if these organizations were recently distinct).	
- Focuses on service delivery partnerships (eg multidisciplinary teams working in primary care) or interprofessional collaboration (eg between clinicians and social workers) without any focus on related collaboration at an organizational level.	
- Focuses on collaborations between organizations within the health care system (eg between primary care practices) or between agencies focused on academic research.	
- Is not a review article or does not include empirical data on collaboration outcomes or processes and mechanisms thought to affect collaboration outcomes. Reviews of partnership models or theoretical frameworks related to partnerships were excluded.	

^a^Depending on local or national context, local government, public health, and social services agencies may deliver some health care or closely related services. Terms for these organizations were therefore included in our literature searches. However, for the purposes of study selection and analysis, these types of organizations and services were not viewed as health care organizations. This means that reviews focused on collaborations between health care and public health, or between health care and social services, were included in the review

The inclusion criteria also focused on excluding studies examining closely related but distinct phenomena—for example, evidence related to service delivery level partnerships (such as multidisciplinary teams), or interprofessional collaboration (for example, between clinicians and social workers), without a clear focus on collaboration at an organizational level.

Titles and abstracts of all papers were screened by a member of the review team to identify relevant studies, with the full text reviewed if it appeared relevant. A 10% sample was screened by a second author, in line with umbrella reviews of a similar scale [55, 56]. Studies were assessed against the inclusion and exclusion criteria. Disagreements about inclusion were resolved by consensus and discussion with a third reviewer if necessary. For the studies included, we assessed risk of bias using the AMSTAR 2 critical appraisal tool [57] (for all studies reviewing quantitative evidence on collaboration impacts) and the Critical Appraisal Skills Programme (CASP) systematic review checklist [58] (for studies that only reported qualitative evidence on factors influencing collaboration functioning).

### Data extraction and synthesis

For included studies, we extracted and summarized data in templates covering the following domains: study design, collaboration contexts, collaboration type or definition, factors influencing collaboration functioning, and collaboration outcomes. We extracted assessments of statistical heterogeneity and pooled effects of impact where meta-analyses were reported. We used Nvivo 12 to help categorize the data and identify themes between the studies. We grouped data on collaboration impacts by type of effects reported (such as evidence of impact on health outcomes or spending). For data on factors influencing collaboration functioning, we used an inductive approach to code the data based on the concepts identified in the studies (such as the role of trust or communication). We developed the code structure iteratively as data were coded and compared [59], and identified overarching themes that linked the concepts identified in the studies (such as factors related to collaboration motivation and purpose). No meta-analysis was carried out as part of the review, given the lack of quality data on health impacts, the broad nature of the phenomena studied, and the heterogeneity of study designs included. We use a narrative approach to reporting the data synthesis [60].

## Results

**Fig. 1:**
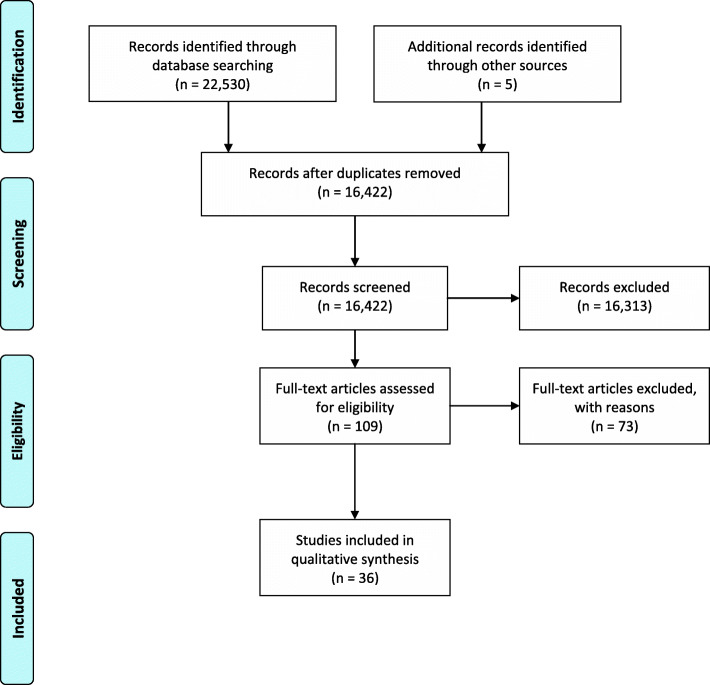
PRISMA flow diagram

Our search identified 16,422 papers, after duplicates were removed. Thirty-six reviews were included in our review (Fig. 1) [4, 23–31, 34, 61–85]. Studies that were reviewed in full but did not meet our inclusion criteria were focused on describing collaboration models or theories [86–100], evidence related to service-level interventions [101–122] or inter-professional collaboration [123–127] without a clear focus on organizational collaboration, collaboration within the health care system [128–132], or collaboration primarily at national or international levels [133, 134]. Other studies were excluded because they were not a review of empirical evidence or the phenomenon reviewed was unclear [135–151], or they repeated or were superseded by another study from the same authors [152–157]. One study could not be obtained [158].

### Collaboration type and context

Table S1 (see Additional File, Table S1) describes the context and type of collaborations in the included studies. The studies reviewed collaborations from a range of contexts. Some studies focused on collaborations in a single country, such as the UK [4, 23, 61, 63–65, 80] or US [28, 72, 79]. The majority of studies included evidence on collaborations from multiple countries and contexts (or did not define the country contexts of the studies reviewed). The definitions of collaboration used in the studies varied widely, as did the types of organizations involved and the aims of the collaborations.

Some studies reviewed collaborations with broad population health goals, such as preventing disease and reducing health inequalities [4, 26–31, 34, 61, 66, 76, 79]. These collaborations often involved health care and social services organizations, public health agencies, and other sectors including housing and education, and more. Other studies reviewed evidence on collaborations with a narrower scope or focus, such as integration between health care and social services [23–25, 62, 63, 68, 80, 84], or care for people with mental health needs [67, 73, 78].

Even within single studies, multiple kinds of collaboration were typically studied together, and interventions were often weakly described. Many reviews combined evidence on collaboration at an organizational-level (such as joint planning or funding of services) with more targeted strategies or interventions that resulted from organizational collaboration (such as care coordination programs for target populations). Evidence related to the impact and functioning of organizational partnerships was therefore hard to disentangle from evidence on related policies and interventions—for example, evidence on specific service-level changes.

### Quality of evidence

Overall, the quality of evidence reviewed was weak (see Additional File, Table S2). The methods of the studies varied, including umbrella reviews, systematic reviews and meta-analyses, scoping reviews, and narrative reviews. Of the reviews that reported data on collaboration impacts, most were deemed to be critically low quality and only three reviews were deemed to be high quality. Of the reviews that only reported data on factors influencing collaboration functioning, most had multiple sources of potential bias—including weak search strategies and limited approaches to assessing and reporting risk of bias in the studies they reviewed. We have not excluded studies from our narrative synthesis based on the quality of the reviews, but we do note limitations or uncertainty in the evidence presented.

### Impacts of collaboration

Twenty-one studies reported on collaboration impacts at a mix of individual and population levels [4, 23–26, 28, 30, 31, 62, 63, 67, 69, 71–74, 77, 78, 81, 82, 84]. They included evidence on health outcomes and health-related behaviours, service access and quality, resource use and spending, and organization or system-level processes related to collaboration. Evidence from the studies on collaboration impacts is summarized in Table S3 (see Additional File, Table S3).

#### Health outcomes

Most studies assessing the impact of collaboration on health outcomes, such as quality of life, mortality or health equity, found no, mixed, or limited evidence of impact. A review and meta-analysis of collaboration between local health and non-health agencies for health improvement found little or no evidence of health benefits [26]. Meta-analysis of effects on mortality, for example, found no effect (relative risk = 1.04 in favour of control, 95% CI 0.92 to 1.17) (see Additional File, Table S3 for effects on morbidity) [26]. A review of public sector collaborations to improve health in targeted communities in England found no evidence of population health improvements [4]. A review of community-level interventions to improve health in the US found insufficient evidence related to population health impacts—though it did find that these interventions could contribute to positive changes in health-related behaviours, such as smoking [28]. Ndumbe-Eyoh and Moffat found mixed impacts on health outcomes and limited evidence on equity impacts of collaboration to improve health for disadvantaged groups [31].

Mason et al. reviewed integrated funding initiatives between health care and social services agencies and found that, in the studies assessing health effects (such as quality of life and mortality), most reported no significant difference compared with usual care [25]. Cameron’s et al’s review of collaboration between health care and social services agencies found no or marginal improvements in health outcomes in studies with comparative designs (some studies with weaker designs reported improvements) [23]. Winters et al. found that most studies did not report positive outcomes (though did not define these outcomes clearly) [24]. And Liljas et al’s review of collaboration to provide more integrated care for older people with multimorbidity found that no studies examining mortality effects reported significant changes in mortality rates [82]. Five reviews found that evidence on health outcomes was limited [63, 67, 69, 74, 84].

Evidence of impact from some kinds of collaborations was more promising. Anderson et al. reviewed evidence on community coalitions to reduce health inequalities among minority groups and concluded that community coalition-driven interventions could benefit minority populations [30]. Community-level system changes—for example, focused on improving housing or green spaces—had little or no impact on measures of health status or health behaviour. But interventions targeting changes in the health and social care system—for example, to improve quality of care—led to small improvements on measures of health status or behaviour in large samples of community residents (though the evidence was rated as very low certainty). More positive impacts were also reported from lay community health worker and group-based health education interventions in large samples of community residents (though, again, the evidence was rated low or very low certainty). Bagnall et al’s review of systems approaches to reducing obesity found that most studies reported some positive effects, including on health-related behaviours and body mass index [81]. One review of collaboration between health and a range of non-health sectors (such education and housing) to prevent and control vector-borne disease also found positive effects in the majority of studies that measured outcomes, including incidence and prevalence of disease [77].

Lopez-Carmen et al’s review of collaboration to improve mental health among indigenous children found few quality evaluations to draw on, but identified some studies reporting positive outcomes among children and their families receiving particular interventions [73]. Similarly, a review of primary care and public health collaboration described weaknesses in the evidence but reported some positive outcomes at an individual and population level, related to chronic disease management, disease control, and maternal child health [71].

#### Service use and quality

Several reviews reported evidence that collaboration in a mix of contexts could improve access to services [31, 62, 71, 73, 77], including for disadvantaged groups [31, 73]. Cooper et al’s review of collaboration in children and young people’s mental health services, however, found more mixed evidence—with some studies suggesting more equitable access and others reporting reductions in access [67]. Some models of financial integration between health care and social service agencies may also have the unintended effect of reducing access for some groups (for example, by creating financial incentives to deny access to more costly patients) [25].

A review of integrated care interventions (including a mix of organizational and service level changes to improve coordination of services) found inconsistent evidence on overall health care utilization and activity, as well as on a range of specific utilization measures (such as clinician contacts and length of stay) [62]. Cameron et al’s review of health and social care collaboration found some evidence that intermediate care could reduce inappropriate admissions to institutional care [23]. Liljas et al’s review of health and social care collaboration found mixed evidence on hospital admissions, readmissions, and length of stay [82].

Evidence of impact on quality of services was mixed. Five reviews reported mixed impacts on quality of care [25, 67, 84] and patient satisfaction [82]. Dowling et al’s review of health and social care partnerships in England found no clear or consistent evidence of improvements in services [63]. Baxter et al. found evidence of improvements in patient satisfaction and perceived quality of care related to integrated care interventions [62]. A review of primary care and public health collaboration also reported some improvements in quality of care [71].

Reviews of some targeted collaboration interventions reported positive impacts. For example, a review and meta-analysis of collaboration to improve child welfare outcomes in the US found that family drug treatment courts were positively associated with entry (odds ratio = 2.94, 95% CI 1.50 to 5.75) and completion (odds ratio = 2.07, 95% CI 1.26 to 3.41) of substance use services, and that family drug treatment courts (odds ratio = 2.40, 95% CI 1.75 to 3.29) and recovery coaches (odds ratio = 1.52, 95% CI 1.17 to 1.99) were associated with increased likelihood of children being reunited with families [72]. Whiteford et al. found that collaboration between mental health care agencies and non-medical supports could increase housing stability, reduce recidivism rates, and improve employment-related outcomes, though also noted that positive outcomes had not been reported in all programs reviewed [78]. The reviews of these interventions, however, were of critically low quality (see Additional File, Table S2).

#### Resource use and spending

Evidence on the resource use and spending impacts of collaboration was limited and mixed. Hayes et al’s review of collaborations between health and non-health organizations found some evidence of increased costs, and—even though economic data were not provided for all studies—‘it was clear that in most studies the interventions required additional resources’ [26].

Two reviews focused on collaborations to deliver more integrated health and care services found inconsistent evidence on costs [62], or weak evidence that did not generally report cost reductions [23]. A review of collaboration between mental health care and non-medical supports found that while some studies reported ‘improved cost efficiency across sectors’, there were also examples of initiatives where costs fell for some partners but increased for others [78].

A review of integrated funding initiatives between health and social care organizations [25] reported impacts on hospital costs and utilization together. It found that most schemes reported mixed (14 schemes) or unclear evidence (5 schemes), around a third of studies found no significant effect on hospital costs or utilization (11 schemes), three schemes reported a reduction in hospital costs or utilization, and one scheme reported increased utilization. Other studies sought evidence on the cost impacts of collaboration but found little data [30, 77, 81, 84].

#### Process impacts

Some reviews reported qualitative evidence on changes in organizational or system-level processes as impacts of collaboration. These included improved collaboration processes and organizational capacity [73, 78], stakeholder buy-in [74], implementation of policies and programs related to health promotion [28], and an increased focus on health inequalities in local plans [4]. Potential negative impacts included increased time commitment and challenges to professional identities [67]. There were also some unintended consequences of financial incentives related collaboration, such as ‘upcoding’ (of ‘nursing home certifiable’ patients in a US demonstration program) [25], cost shifting [78], and skewing local priorities [71]. More broadly, qualitative evidence on factors shaping collaboration functioning—outlined in the following section—describe various processes that can support or constrain joint working.

### Factors influencing collaboration functioning

**Fig. 2:**
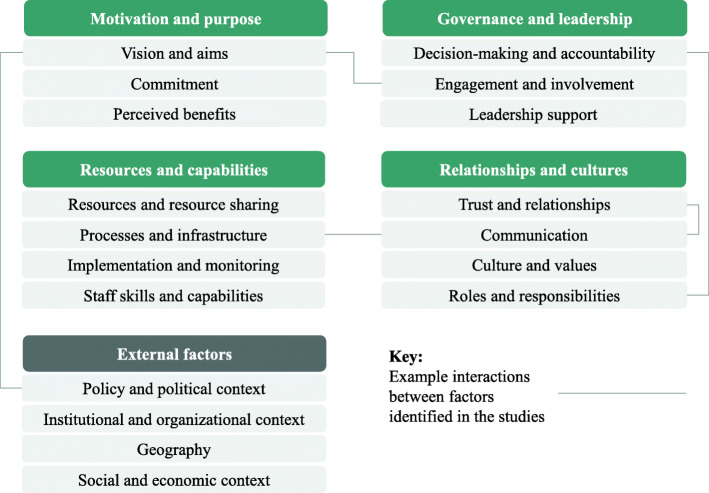
Factors influencing collaboration functioning and example interactions between them Notes: The interactions between factors are examples identified in the studies reviewed. They are not an exhaustive list of all interactions between the factors identified. The relationships may move in both directions (eg involving staff may help create a shared vision, while having a shared vision may help with the task of engaging other partners), and may support or constrain collaboration in different contexts (eg national policies can help or hinder)

Twenty-nine studies reported on factors shaping the success and functioning of organizational collaboration (see Additional File, Table S4). This included factors related to collaboration aims and motivation, resources and capabilities, cultures and relationships, governance and leadership, and external context (Fig. 2). The factors overlap and interrelate, and sometimes come into conflict.

#### Motivation and purpose

Organizations collaborated for different reasons, which shaped how they worked together. A shared vision between local organizations and clear aims for collaboration were commonly identified as factors contributing to partnership success [23, 27–29, 34, 63, 64, 66, 69, 74, 76–78, 80, 81, 83, 85]. Involving organizational staff was identified as one route to creating a shared vision [23, 28, 66, 76]; and a shared vision, in turn, may help with the task of engaging other partners [28]. On the flipside, unclear or unrealistic aims, competing agendas, and uncertain benefits were all identified as factors that can hold back organizational collaboration [23, 61, 64, 67–69, 71, 76, 77].

National policies supported local partnerships to emerge in various contexts studied (see section on external context) [27, 71, 76, 80, 83, 85]—though some studies also noted that national government policies mandating local collaboration may reflect an underlying lack of motivation for joint working among local agencies, and could create conditions for future conflict [65, 85]. Commitment to collaboration from local leaders and staff was commonly thought to be needed for partnerships to work effectively [28, 29, 34, 66–70, 77, 79].

#### Relationships and cultures

Multiple studies described how collaboration was more likely to be successful if partners trust each other [23, 63, 67, 68, 70, 71, 81, 84, 85] and have positive relationships [23, 25, 29, 64, 67, 73, 74, 77, 79, 81, 83]. For example, Davies et al. identified lack of trust between health care staff and care homes as a barrier to integrated working [84]. Historic relationships between agencies—present or absent; good or bad—shaped how local partnerships developed and functioned [23, 24, 28, 34, 66, 68, 69, 74, 77, 85].

Relationships were also affected by cultural and professional differences between agencies and staff within them—often identified as barriers to collaboration [23, 25, 65, 67–69, 76–78, 85]. In some cases, shared values could bring local agencies together—for example, united by a commitment to good governance or reducing health inequalities [71, 81]. But differences in values could also fundamentally undermine collaboration efforts. Williams, for example, found that philosophical differences between health and social care and criminal justice agencies—between ‘care and control’ sectors—contributed to various structural and procedural challenges experienced among crime prevention and reduction partnerships in England [65].

Clarity on roles and responsibilities of different agencies was thought to help collaborations make decisions, implement programs, and function effectively [23, 24, 66, 67, 69, 71, 74–76, 78, 85]. Lack of clarity could lead to protectionism, concerns about loss of power, and underuse of particular skills or services within the partnership [23, 64, 75]. For example, Green et al. described how lack of understanding of aboriginal health workers among public service agencies contributed to their underutilization within partnerships to improve care for indigenous children [75]. Developing clear frameworks and processes for collaboration [23, 29, 64] and joint training for staff between agencies [69] (see section on resources and capabilities) were both identified as mechanisms that could help improve clarity on organizational roles. Yet role clarity may not be needed for all kinds of partnerships, or at all levels within them. Corbin et al. noted that flexibility on roles may help partnerships be more inclusive and garner increased resources [34]. And, at a service level—for example, for staff delivering programs within the partnership—flexibility may be needed to support multidisciplinary teams to function [23].

How and when partners communicate was widely thought to affect how collaborations work [23, 24, 27, 29, 34, 61, 65–71, 73–79, 83, 85]. The simple interpretation from the literature is that good communication helps, while poor communication makes things harder. Good communication was thought to be open and frequent (though partners may disagree about how and when communication should happen [34]) [24, 66, 71, 74, 79, 85], and involve sharing of information and best practice [27, 61, 73, 83]. Various mechanisms were identified to help agencies do this (see section on resources and capabilities), such as regularly scheduled meetings and protocols for information sharing [24, 74]. But communication issues within the partnerships studied were widespread, exacerbated by lacking or incompatible information systems, conflicting procedures, lack of trust between organizations and professions, and more [23, 61, 65, 67–69, 78]. The quality of communication was thought to affect various other factors shaping collaboration success, such as trust and understanding between partners [24, 66, 70, 71, 75].

#### Resources and capabilities

Organizational collaborations depended on having sufficient resources to fund and deliver interventions, such as new service models or programs [28, 29, 34, 66, 67, 69–71, 73, 74, 76, 80, 81, 83, 85]. Lack of resources for joint working—funding, staff, equipment—was identified as a common barrier to collaboration [23, 34, 61, 67–69, 71, 73, 76–78, 85], and could result in increased staff workload [75, 80]. Short-term or uncertain funding also held back some collaborations [61, 64, 69, 71].

Sharing resources between agencies—for example, through pooled budgets—was identified as one mechanism that may facilitate joint working [23, 61, 69, 71, 83], and, in some cases, as a route to accessing additional resources [83]. But studies also found that sharing resources could lead to challenges in ensuring equitable funding between agencies [24, 25, 69, 71, 85], and could create fears of cost-shifting among some partners [68, 85]. Ultimately, having resources is not enough: resources also needed to be used effectively by local agencies to generate positive impact [74, 79].

The ability of organizations to collaborate was shaped, in part, by the infrastructure in place between them. Shared processes and systems—such as agreements for sharing information, joint meetings, and planning processes (see section on governance and leadership)—were thought to support organizations to communicate and work together [24, 29, 34, 64, 67, 70, 71, 73, 74, 80, 83]. Several studies suggested that co-location or close proximity of teams may support joint working [34, 67, 71, 78, 80, 83]—though this appears to relate largely to teams delivering services. Health impact assessments—a mix of methods and tools to help identify the health and equity impacts of a particular policy or program—were also identified as a key mechanism for local governments and other partners seeking to promote intersectoral action [27, 76]. On the flipside, fragmented or conflicting processes between agencies could hold back local partnerships [24, 25, 65, 67, 68, 70, 71, 77, 85]. Collaborations also needed skills to plan and implement their chosen interventions [28, 29, 69, 74, 79, 85], and monitor and evaluate the results [27, 28, 34, 63, 69, 74, 76–78, 81].

The skills and capabilities of staff also shaped collaboration functioning. Staff able to work across organizational and professional boundaries—sometimes referred to as ‘boundary spanners’—were thought to contribute to partnership success [24, 34, 64, 67, 71, 75, 85]. Staff training on collaboration processes and joint training between agencies were thought to help improve collaboration and understanding between sectors [23, 24, 28, 64, 67, 69–71, 74–76, 79, 83, 84], while high staff turnover or lack of continuity of key staff could hold back collaboration [24, 34, 69, 70, 73, 84].

#### Governance and leadership

Differences in decision-making processes, competition for power and resources, and lack of accountability between agencies were identified as barriers to collaboration [25, 66, 68, 70, 71, 76, 77, 85]. As a result, clear decision-making and accountability arrangements were thought to contribute to collaboration success [34, 63, 64, 66, 78, 85]. The literature is not particularly clear what this means in practice. Example mechanisms included conflict-resolution processes [78, 79, 83], such as a neutral convener [83], and formalized rules or contracts between agencies [71, 79].

The literature is more clear, however, that good governance means involving all relevant agencies and stakeholders [24, 27, 28, 34, 68, 70, 71, 74, 77–79, 81, 85]. This includes front-line staff [24, 64, 69, 71]. But broad membership could also bring challenges for decision-making, such as limiting the chance of consensus [66]. Multiple studies identified the importance of community involvement—including direct involvement of community members and community-based organizations—for collaborations to be successful [24, 28, 63, 66, 71, 81]. This may help ensure that organizations understand community needs and design appropriate interventions [66, 71, 73, 81].

Senior leaders played a key role in shaping how local collaborations and their governance functioned—for better or worse [24, 28, 29, 34, 63, 66–70, 74, 76–81, 85]. Leadership commitment was thought to be needed for collaborations to work (see section on motivation and purpose). Leaders used their power to free up resources for joint working [34, 70, 76], help resolve conflicts [29, 78], promote openness and information sharing between agencies [34, 74], and more. But leaders could also block partnership working by defending territorial or organizational interests [68, 70]. The collective involvement of organizational leaders—beyond their individual impact—was thought to contribute to partnership effectiveness [24, 28, 34, 66].

#### External factors

Collaborations do not exist in a vacuum. While the internal characteristics of partnerships—their leadership, governance, composition, and so on—mattered, the broader context in which local agencies operated shaped how they worked together and the impact they could achieve.

National policy context influenced the local partnerships reviewed. On the one hand, national policies promoted or incentivized joint working in several contexts studied [27, 65, 71, 75, 76, 80, 83, 85]. Government policies on tackling health inequalities, for example, facilitated local partnerships to develop in Europe and elsewhere [27, 76]. In some contexts, such as the UK, national policymakers also mandated partnership working between agencies—though some studies suggested that doing so risks lowering the chances of partnership success, including by undermining the time needed to develop local relationships [66, 85]. On the other hand, some studies suggested that national policy priorities could dampen or conflict with local priorities [76, 85]. And constantly shifting national policies may confuse or undermine local partnerships—as was experienced by those involved in area-based partnerships between health care, social services, and other sectors in England in the late 1990s and early 2000s [61]. Policies not directly focused on collaboration—for example, payment systems in the health care sector—could also create barriers to collaboration between health and social care [80].

The institutional and organizational context of health care, social services, and other sectors also shaped local collaborations [25, 63, 68, 75]. For example, Mason et al. identified differences in national performance systems, pension schemes, and employment arrangements as barriers to collaboration between health and social services in the UK [25]. Several studies also noted that organizational restructuring could hold back local collaboration [23, 61, 69, 71]—for example, by creating uncertainty among agencies [71] and requiring leaders to renegotiate relationships [61].

Other external factors identified in the literature included political context [27, 34, 77]—for instance, ‘political will’—and the geographical location and boundaries of collaborating agenices [61, 69, 77, 85]. Finally, the social and economic context within which partnerships operate was recognized as a factor shaping collaboration functioning potential and impact [28, 34, 76].

## Discussion

Collaboration between health care, social services, and other sectors is often seen as a common-sense route to improving population health. We sought to review evidence on the health impacts of collaboration between local health care and non-health care organizations, as well as the factors shaping their functioning and success. We identified 36 studies that reviewed evidence on local collaborations in various contexts and synthesized the results.

Overall, there is little convincing evidence to suggest that collaboration between local health care and non-health care organizations improves health outcomes. Evidence of impact on health services is mixed—though some studies suggest collaboration may improve access to services, and one high quality review found that integrated care interventions may improve patient satisfaction [62]. Evidence on resource use and spending was limited and mixed. Across the studies reviewed, positive impacts appear more likely to be reported for more targeted interventions (for example, health system and community outreach interventions reviewed by Anderson et al. [30]) or narrow measures of impact (such as access). Where meta-analyses indicated positive impacts, there was generally substantial heterogeneity. The quality of evidence reviewed was generally weak and the types of collaborations studied varied widely.

There may be several explanations for the lack of evidence on impact. On the one hand, the emperor may simply have no clothes: collaboration between health care and non-health care organizations may not deliver the kinds of impacts that many policymakers expect. On the other, collaborations may be fiendishly difficult to do—as illustrated by the many barriers to joint working identified in the literature—so while effective partnerships may contribute to better health, implementation issues render them rare. A further explanation is that the effects of collaboration are difficult to measure. Evaluating these kinds of collaborations brings significant methodological challenges [86, 159]—given that they are hard to define, involve multiple organizations and interventions spread over space and time, have diverse and often long-term aims, and operate alongside many other factors that affect health. This is particularly the case for collaborations focused on broad population health improvements. The impact of local collaborations may be positive, but modest—and easily drowned out by the combination of other factors influencing the population’s health. A mix of these explanations may be true—with benefits overestimated, hard to deliver, and hard to measure.

Many studies report on factors and mechanisms associated with better or worse collaboration. We grouped these into five domains—covering motivation and purpose, relationships and cultures, resources and capabilities, governance and leadership, and external factors. These factors offer pointers for practitioners and policymakers seeking to foster collaboration, as well as examples of issues faced in various contexts. Several factors, such as quality of communication between partners and availability of resources, appear consistently across multiple studies. But without better evidence on the impact of different collaborative efforts, it is difficult to know how and whether these and other factors actually shape collaboration outcomes. There are also limited data on the interaction between factors, their relative importance in different contexts, and the conflicts and trade-offs between them. As a result, we know little about which kinds of collaborations work, for whom, and in what contexts.

Despite this, collaboration between local agencies to improve health looks here to stay. Faith in collaboration has been a driver of health policies in various countries over decades—and currently forms part of health system reforms in the US, UK, and elsewhere [7, 37, 39, 40]. COVID-19 may stoke policymakers’ belief in collaboration even further. The Secretary of State for Health and Social Care in England, for example, has described the positive impact of collaboration between local services as a core lesson from the COVID-19 response, and identified increased collaboration between the NHS, local government, and wider public services as a policy priority as the country recovers from the pandemic [160]. Legislation has been proposed to formalize existing health and social care partnerships in England [161]. The evidence reviewed here suggests that policymakers and local leaders should be realistic about the kind of impacts that collaboration may deliver on its own. The potential benefits of closer working between health care and social services agencies—for example, on preventing health service use and reducing costs—have often been overstated [37, 162]. And the various cultural, institutional, practical, and other issues that hold back collaboration risk being underplayed.

Ultimately, local collaborations are shaped by the broader social, political, and economic structures in which they operate. Better communication, say, may help agencies coordinate local health interventions. But broader state and national policy decisions—for example, government policies on the level and distribution of spending on income support, education, and social services—will fundamentally shape health and health inequalities in those communities [7, 51, 163]. Local collaborations must therefore be understood within their broader political context, and alongside other interventions that interact to shape population health.

Conceptualizing collaborations as one component in a complex system may help us better understand their potential contribution to improving health. Take health partnerships in England under the New Labour governments (1997 to 2010) as one example. Various ‘area based’ collaborations between health care, social services, and other agencies were developed in England in the late 1990s and 2000s as part of a broader national strategy to reduce health inequalities between richer and poorer areas. The strategy evolved over time and involved a range of interventions—including better support for families, engaging communities, efforts to tackle poverty, improving NHS prevention and treatment, and a mix of other policy measures, combined with increased investment in the NHS, social care and other services [164–166]. Evaluations of the local collaborations developed during this period found no clear evidence of their effect on health outcomes [4, 61]. But more recent evidence suggests that the broader government strategy may have been partially effective in reducing health inequalities over time—associated with reductions in regional inequalities in life expectancy and infant mortality [167, 168]. Local collaborations may have contributed to a complex system of interventions affecting health, operating at multiple levels. For example, local collaborations were one mechanism supporting the delivery of potentially powerful policy interventions introduced by government, such as additional spending on the NHS and social programs.

Disentangling the distinctive impact of local collaborations from the broader context in which they operate will remain a challenge for researchers. But some methods may help identify features of collaboration that have the potential to contribute to better health in different contexts. Positive deviance sampling [169, 170], for example, is based on the assumption that elements of ‘what works’ can already be found in organizations or communities that consistently experience better performance on selected indicators. Feasible solutions to complex problems may be identified by studying these cases. Positive deviance sampling is increasingly used in health services research to identify approaches for improvement—including Brewster et al’s study of collaboration among health care and social service agencies in areas that achieve relatively low health care utilization and costs for older adults in the US [171]. This kind of approach might be utilized in other contexts to help understand whether organizations in communities with better population health have distinct patterns of cross-sector collaboration.

### Limitations

This study has several limitations. First, the kinds of collaboration described in the literature are often broadly defined and weakly described. Information on the form of collaboration—for example, which agencies work together and how—is often limited. And multiple types of collaboration are often studied together, making the evidence hard to disentangle. Our review excluded studies that focused on collaboration between professionals or services—for example, through multi-disciplinary teams at a service level—without a clear focus on collaboration at an organizational level. But some studies that we included reviewed evidence on a mix of collaboration interventions—not all directly related to our phenomena of interest.

Second, our search strategy focused on identifying evidence on collaboration between health care and non-health care organizations broadly speaking—with terms like collaboration, coalition, and partnership, alongside terms related to health and social services organizations and inter-organizational working (see Additional File 1). This broad approach is a strength of the review, given that it identified a large body of relevant literature on collaboration between agencies to improve health. But it also means that reviews of interventions involving organizational collaboration but not using these terms—for example, evidence on pooled financing models to fund local health interventions—may not have been identified fully through our searches.

Third, we only synthesized evidence from reviews of the literature. This allowed us to make sense of a large body of diverse evidence. But it is likely that some relevant primary studies have not been included in our review. It means that some studies may be duplicated between reviews—though this is unlikely to skew our findings, given the lack of convincing evidence overall, and the fact that we did not undertake a pooled quantitative analysis of collaboration impacts. It means that a heterogenous mix of interventions and contexts were studied together. Our study design—two steps removed from the primary evidence—also means that the context and richness of the original primary data are largely lost in our review. The exclusion of non-English language papers will have also affected the studies we identified.

Finally, our study is limited by the quality of evidence reviewed. The reviews included in our study were typically poor quality, and themselves often cited the limitations of the primary studies they reviewed. Weak descriptions of the factors shaping collaboration functioning make it difficult to identify the mechanisms that might help collaboration efforts in different contexts. Nonetheless, the evidence reviewed provides useful pointers for policy and practice.

## Conclusion

Collaboration between health care, social services, and other sectors is widely promoted as a route to improving population health. Theory suggests that collaboration might help local organizations combine their skills and resources to better meet community needs. But competing institutional norms and priorities may also create conditions for conflict. We found little convincing evidence to suggest that collaboration between local health care and non-health care organizations improves health outcomes. The literature offers pointers for policymakers and practitioners on factors thought to be associated with better or worse collaboration. But, overall, we know little about which collaborations work, for whom, and in what contexts. Local collaborations should be understood within their broader political context, and alongside other interventions and factors that interact to shape population health.

## Supplementary Information

**Additional file 1.** Medline search strategy

**Additional file 2: Table S1.** Study context and collaboration type

**Additional file 3: Table S2.** Study quality assessments

**Additional file 4: Table S3.** Summary of evidence on collaboration impacts

**Additional file 5: Table S4. **Summary of evidence on factors influencing collaboration functioning

## Data Availability

The full database search results retrieved during the study are available from the corresponding author on reasonable request. The list of papers that were screened in full and the list of papers that were analysed in the review are included in the published article.
