# Supplementary material for: The impacts of collaboration between local health care and non-health care organizations and factors shaping how they work: a systematic review of reviews
Source: BMC Public Health. 2021 Apr 19;21:753. doi: 10.1186/s12889-021-10630-1 (PMC8054696; doi:10.1186/s12889-021-10630-1)
Supplement: Supplementary file 1 — Additional file 1. Medline search strategy [file 12889_2021_10630_MOESM1_ESM.docx]

**Additional File 1: Medline search strategy**

Database: Ovid MEDLINE(R) <1946 to December Week 1 2019>

--------------------------------------------------------------------------------

1 ((collaborat* or partners* or alliance* or coalition* or network* or joined-up or coordinat* or integrat* or joint-working or cooperat*) adj4 (organisation* or organization* or inter-organisation* or inter-organization* or agenc* or multi-agency or institution* or cross-sector* or multi-sector* or multisector* or inter-agency or interagency or intersector* or interinstitution* or health care or healthcare or health system* or NHS or health service* or hospital* or primary care or general practi* or community service* or community health service* or mental health or public health or local government or social care or social service*)).ti,ab. (55828)

2 Health Care Coalitions/ (2337)

3 Intersectoral Collaboration/ (1699)

4 Cooperative Behavior/ (42493)

5 Interinstitutional Relations/ (10581)

6 (health or outcome* or quality or equity or inequit* or inequalit* or mortality or morbidity or prevent*).ti,ab. (4385986)

7 Health Equity/ (940)

8 review.ti,ab. (1249016)

9 "Systematic Review"/ (116819)

10 1 or 2 or 3 or 4 or 5 (103911)

11 6 or 7 (4386064)

12 8 or 9 (1253693)

13 10 and 11 and 12 (6240)

14 limit 13 to (english language and yr="1999 -Current") (5454)

***************************
