# Supplementary material for: The impacts of collaboration between local health care and non-health care organizations and factors shaping how they work: a systematic review of reviews
Source: BMC Public Health. 2021 Apr 19;21:753. doi: 10.1186/s12889-021-10630-1 (PMC8054696; doi:10.1186/s12889-021-10630-1)
Supplement: Supplementary file 2 — Additional file 2: Table S1. Study context and collaboration type [file 12889_2021_10630_MOESM2_ESM.docx]

**TABLE S1: study context and collaboration type**

| **Study** | **Context** | **Collaboration type** |
| --- | --- | --- |
| Anderson et al (2015). Community coalition‐driven interventions to reduce health disparities among racial and ethnic minority populations^[[1]](#endnote-1)^ | - Community coalitions with at least one racial or ethnic minority group representing the target population, and at least two community public or private organizations - Studies included focused on academic and community partnerships, partnerships between public health and other agencies, community-based agency partnerships - Health or health care agency was lead sector in 13 studies | - Community coalitions, defined as ‘conglomerates of citizen groups, public and private organizations, and professions that are characterized by representation from multiple community sectors in bottom‐up planning and decision making. They operate through partnerships and emphasize using local assets and resources to build community capacity. The focus of a community coalition may vary depending on the sectors of the community involved (eg education, public safety, public health)’ - Four types of interventions used by coalitions: broad‐scale community system‐level change (eg improving housing or green spaces), broad‐scale health or social care system‐level change (eg to improve quality of care), lay community health outreach workers (eg to promote behaviour change), group‐based health education and support for targeted groups (eg diabetes) |
| Andersson et al (2011) Organizational approaches to collaboration in vocational rehabilitation-an international literature review^[[2]](#endnote-2)^ | - Vocational rehabilitation, defined as ‘a multidisciplinary intervention to help individuals to return to work after an occupational injury, or a period of unemployment or sickness’ - Typical partners include health care and social services, occupational health services, employment services, and social or private insurance - Studies from Sweden, Canada, US, Netherlands, UK, Australia, Belgium, Norway | - Seven models of collaboration in studies reviewed, often in combination: information exchange, case coordination, interagency meetings, multidisciplinary teams, ‘partnership’ (‘formal agreements between two or more organizations to integrate their services across organizational boundaries), co-location, budget pooling |
| Auschra C (2018). Barriers to the integration of care in inter-organisational settings:  a literature review^[[3]](#endnote-3)^ | - ‘Inter-organizational collaborations’ to support integrated care - Focus on ‘health service delivery’ (not defined). Studies largely focus on health care and social services | - Inter-organizational collaborations, defined as ‘dyadic relationships between two partner organisations or as inter-organisational networks, implying relationships between at least three partners’ - Integrated care defined as ‘a coherent set of methods and models on the funding, administrative, organisational, service delivery and clinical domains designed to create connectivity, alignment and collaboration within and between the cure and care sector’ |
| Bagnall et al (2019). Whole systems approaches to obesity and other complex public health challenges: a systematic review^[[4]](#endnote-4)^ | - Systems approaches to obesity - Studies from US, Canada, UK, Europe, and others involved in the WHO Healthy Cities Network - Interventions targeted a mix of population groups, including adults, children, deprived areas, minority groups, socially excluded groups, people with disabilities - Partnerships poorly described. Partners include local government, health care, schools, community groups, childcare, and others | - Whole systems approaches to obesity, defined as ‘those that consider the multifactorial drivers of overweight and obesity, involve transformative co-ordinated action across a broad range of disciplines and stakeholders, operate across all levels of governance and throughout the life course’ - Heterogenous and wide-ranging interventions |
| Baxter et al (2018). The effects of integrated care: a systematic review of UK and international evidence^[[5]](#endnote-5)^ | - Studies of integrated care focused on a range of population groups, most commonly older people - Studies from the UK, US, Canada, Australia, Netherlands, Sweden, Spain, Germany, Switzerland, France, Norway, Finland, New Zealand, Austria | - Integrated care, defined as ‘changes to health or both health and health-related service delivery which aim to increase integration and/or coordination’ - Interventions with four broad elements, often in combination: patient care interventions, changes to organizations and systems, changing staff or employment arrangements, changes to finance or governance arrangements |
| Cameron et al (2014). Factors that promote and hinder joint and integrated working between health and social care services: a review of research literature^[[6]](#endnote-6)^ | - ‘Jointly organised’ services for older people and people with mental health conditions in the UK | - Studies included focused on service delivery partnerships, ‘structurally integrated services’, and pooled budgets |
| Cooper et al (2016). Interagency collaboration in children and young people's mental health: a systematic review of outcomes, facilitating factors and inhibiting factors^[[7]](#endnote-7)^ | - ‘Interagency collaboration’ across child and young people's mental health services - Sectors include child and adolescent mental health services, school-based providers, child welfare, counseling - Studies from the UK, North America, Scandinavia, Australia | - Interagency collaboration, defined as ‘the process in which different professional services work together to try and positively impact care’ - Partnerships involved two or more agencies or professional groups |
| Corbin (2016). What makes intersectoral partnerships for health promotion work? A review of the international literature^[[8]](#endnote-8)^ | - ‘Intersectoral partnerships’ focused on health promotion - Partnerships focused on cancer, violence prevention, HIV/AIDS, nutrition labeling, physical activity - Studies from the US, Ireland, Tanzania, Canada, Netherlands, Australia, UK | - Partnership defined as ‘any arrangement in which people and/or organizations join together to promote health’ |
| Davies et al (2011). A systematic review of integrated working between care homes and health care services^[[9]](#endnote-9)^ | - Integrated working between primary health care and care homes for older people - Studies from UK, Australia, USA, Sweden | - Integration between health and care services, defined as micro level collaboration (eg staff working together), meso level collaboration (eg organizational structures to support teams to work together), and macro level collaboration (eg joint funding for health care and care homes) - Studies included examples of micro, meso, and macro integration |
| Dowling et al (2004). Conceptualising successful partnerships^[[10]](#endnote-10)^ | - Health and social care partnerships in England - Wider policy context of New Labour's focus on partnership working in the delivery of health care, social services and other public services | - Partnerships defined as ‘a joint working arrangement where partners are otherwise independent bodies cooperating to achieve a common goal; this may involve the creation of new organizational structures or processes to plan and implement a joint program, as well as sharing relevant information, risks and rewards’ |
| Errecaborde et al (2019). Factors that enable effective one health collaborations: a scoping review of the literature^[[11]](#endnote-11)^ | - Focuses on ‘One Health’ collaborations, defined as ‘the integrative effort of multiple disciplines working to attain optimal health for people, animals, and the environment’ - Collaborations in response to infectious disease-related events - Studies from Europe, the Americas, Asia, Africa, Oceana, Middle East | - Collaboration defined as two or more sectors working together - Focused on both preparedness (eg planned or ongoing work) and responsive (eg emergency health events) collaborations |
| Foster-Fishman et al (2001). Building collaborative capacity in community coalitions: a review and integrative framework^[[12]](#endnote-12)^ | - Community coalitions focused on improving health and wellbeing - Context not described, but article is US-focused | - Community coalitions not defined - Review focused on ‘all forms of collaborative venues including task forces, community coalitions, multiple stakeholder groups, interagency coordinating councils, and coordinating communities’ |
| Gannon-Leary et al (2006). Collaboration and partnership: A review and reflections on a national project to join up local services in England^[[13]](#endnote-13)^ | - Partnership working between public services in England - Review part of a wider evaluation of the Framework for Multi-Agency Environments project (a UK government initiative focused on multi-agency information sharing at a local authority level to improve services) | Collaboration defined in two ways:   - ‘a mechanism for developing a multi-agency partnership strategy in which partners work together towards a common set of goals’ - ‘the function of exchanging information, altering activities, sharing resources and developing the capacity of another organization or individual for mutual benefit in order to achieve a common aim’ |
| Green et al (2014). Cross-sector collaborations in Aboriginal and Torres Strait Islander childhood disability: A systematic integrative review and theory-based synthesis^[[14]](#endnote-14)^ | - Collaborations among services for Aboriginal and Torres Strait Islander children with a disability and their families - National policy context of Australian government initiatives to coordinate public services for this group | - Focus on ‘collaboration or interaction within or across two or more providers/sectors’ - Collaboration models included collaboration within the health sector, between health and education sectors, and in schools or early childhood development centers - Most studies focused on hearing impairment and learning disabilities |
| Guglielmin et al (2018). A scoping review of the implementation of health in all policies at the local level^[[15]](#endnote-15)^ | - Local health in all policies approaches, defined as ‘an approach to public policies across sectors that systematically takes into account the health implications of decisions, seeks synergies, and avoids harmful health impacts in order to improve population health and health equity’ - Studies from 14 countries, most commonly Sweden, Australia, Canada, Finland, Netherlands, Norway | - Studies focused on ‘multiple government sectors (and may or may not include the private sector) collaborating (working together in some capacity)' - Local partnerships at a ‘city or municipal’ level |
| Hayes et al (2012). Collaboration between local health and local government agencies for health improvement^[[16]](#endnote-16)^ | - ‘Interagency collaboration’ and partnership between statutory health and local government agencies with interventions aimed at improving health | - Collaboration defined as ‘two or more parties that pursue an agreed set of goals and work cooperatively toward a set of shared health outcomes' - Collaborations focused on care for individual patients through multi-disciplinary teams, population level health promotion or disease prevention, mental health, chronic disease management, healthy lifestyles, frail elderly |
| Herdiana et al (2018). Intersectoral collaboration for the prevention and control of vector borne diseases to support the implementation of a global strategy: a systematic review^[[17]](#endnote-17)^ | - Collaboration to support vector-borne disease management - Studies from Americas, Western Pacific, South East Asia, Africa - Collaborations involving 26 sectors, including health, education, housing, immigration, child and women welfare, rural development, and others | - Intersectoral collaboration, defined as ‘a recognised relationship between health sector and another sector to take action on an issue to achieve health outcome to be more effective, efficient or sustainable’, and ‘cooperation between different sectors of society such as the public sector, civil society and the private sector’ - Interventions primarily sub-national (10 studies focused on national or broader collaborations), and most commonly focused on community or health education |
| Liljas et al (2019). Impact of integrated care on patient-related outcomes among older people: a systematic review^[[18]](#endnote-18)^ | - Integrated health and social care for older adults with multimorbidity - Studies from North America and Western Europe | - Integrated care, defined as ‘a coherent set of methods and models on the funding, administrative, organisational, service delivery and clinical levels designed to create connectivity, alignment and collaboration within and between the cure and care sectors’ - Studies focused on ‘organisational level’ integration (eg collaboration between health and social care agencies) and ‘system level’ integration (eg planning, purchasing, and other activities across the system) |
| Lopez-Carmen et al (2019) Working together to improve the mental health of indigenous children: A systematic review^[[19]](#endnote-19)^ | - Collaboration focused on improving children’s mental health among indigenous children in Canada, Australia, New Zealand, Norway, US - Sectors considered were primary health care, specialist mental health, education, child protection, criminal justice | - Intersectoral service integration, defined as ‘individual, organizational, or inter-organizational levels of collaboration or coordination between [primary health care] and a categorically different education, mental health, juvenile justice and/or child protection service to provide more comprehensive support to address mental health or an (explicitly identified) determinant of Indigenous children's mental health’ - Main strategies for service integration: community health workers, multidisciplinary teams, staff and organizational capacity building, community engagement, empowering families, counselling, adaption of care to address cultural factors, strengthening culture and identity |
| Mackie and Darvill (2016). Factors enabling implementation of integrated health and social care: a systematic review^[[20]](#endnote-20)^ | - Integration of health and social care services in the UK - Studies focus on integrated care in community settings, targeting people with long term conditions | - Integrated care, defined as ‘an organising principle for care delivery to improve patient care and experience through improved coordination’ |
| Martin-Misener et al (2012). Strengthening Primary Health Care through Public Health and Primary Care Collaborations Team. A scoping literature review of collaboration between primary care and public health^[[21]](#endnote-21)^ | - Collaboration between primary care and public health - Studies from the US, UK, other Western European countries, Canada Australia, New Zealand | - Collaborations aimed at improving health care by coordinating services, improving access to care, applying a population perspective to medical practice, using clinical practice to identify and address community health problems, strengthening health promotion and health protection by mobilizing community campaigns, and collaborating around policy, training and research |
| Mason et al (2015). Integrating funds for health and social care: an evidence review^[[22]](#endnote-22)^ | - Integration of finances between health and social care organizations - Studies from England, Scotland, Northern Ireland, Canada, Australia, US, Sweden | - Eight types of integration: transfer payments (eg one authority contributes to the budget of another), cross-charging (eg compensating one sector for failures in another), aligned budgets (eg partners use own resources for joint objectives), lead commissioning (eg one agency leads purchasing based on joint aims), pooled funds (both agencies pay into joint fund for agreed aims), integrated management/provision with pooled funds (eg partners pool resources and staff), structural integration (eg finances integrated under single agency), lead commissioning with aligned incentives (eg pay for performance schemes) |
| Ndumbe-Eyoh and Moffat (2013). Intersectoral action for health equity: a systematic review^[[23]](#endnote-23)^ | - ‘Intersectoral action’ initiatives in the US, UK, Canada, Australia, New Zealand - Most studies focused on local or community initiatives. All interventions focused on disadvantaged groups | - Intersectoral action, defined as ‘intersectoral interventions, policies and programs, undertaken by the public health sector in collaboration with governmental and non-governmental sectors outside of health’ - Interventions categorized into three groups: upstream (eg improving housing conditions), midstream (eg food security), downstream (eg care coordination) |
| Ogbonnaya and Keeney (2018). A systematic review of the effectiveness of interagency and cross-system  collaborations in the United States to improve child welfare outcomes^[[24]](#endnote-24)^ | - Collaboration between agencies to improve child welfare outcomes - All studies focused on substance use and included participants in the US - Legislation in the US mandates that agencies collaborate to deliver outcomes related to child safety, permanency, and wellbeing | - Studies included categorized as ‘coordination’ (‘more formalized joint working, but no sanctions for non-compliance’) and ‘integration’ (‘organizations merge to create new joint identity') - Interventions involved a team of individuals (eg families, treatment providers, mentors, and the courts) to address caregivers' substance use and improve child welfare outcomes |
| Perkins et al (2010). ‘What counts is what works’? New Labour and partnerships in public health^[[25]](#endnote-25)^ | See Smith et al (2009) | See Smith et al (2009) |
| Rantala et al (2014). Intersectoral action: local governments promoting health^[[26]](#endnote-26)^ | - ‘Intersectoral action’, including issue-specific approaches (eg to reduce obesity) and broader health goals - 25 local government cases in 19 countries from the WHO regions of the Americas, Eastern Mediterranean, Europe, South-East Asia and Western Pacific - Population size ranges from less than 50,000 to around 10,000,000 | - Intersectoral action (ISA), defined as ‘how the health sector works with other governmental and non-state sectors to improve health and well-being, address the complex risk factors for health and ensure full access to health and health equity’ |
| Roussos and Fawcett (2000). A review of collaborative partnerships as a strategy for improving community health^[[27]](#endnote-27)^ | - Community level interventions involving ‘collaborative partnerships’ to improve health in the US - Collaborations focused on a wide range of health issues, including substance use, adolescent pregnancy, cardiovascular disease, crime and violence, health services, HIV/AIDS, immunization, infant mortality, lead poisoning, nutrition, food security | - Collaborative partnerships, defined as ‘an alliance among people and organizations from multiple sectors, such as schools and businesses, working together to achieve a common purpose. In public health, collaborative partnerships attempt to improve conditions and outcomes related to the health and wellbeing of entire communities’ |
| Savic et al (2017). Strategies to facilitate integrated care for people with alcohol and other drug problems: a systematic review^[[28]](#endnote-28)^ | - Integrated care for people with alcohol and drug problems - Studies from North America, Europe, Australia | - Integrated care, conceptualized as coordination between alcohol and drug services, and coordination between alcohol and drug services and non-alcohol and drug services, such as mental health, community health, and housing - Most studies focus on strategies to integrate services between alcohol and drug services and non- alcohol and drug services |
| Seaton et al (2018). Factors that impact the success of interorganizational health promotion collaborations: a scoping review^[[29]](#endnote-29)^ | - Collaboration for health promotion involving two or more partners - Studies from the US, Canada, Australia, Denmark, Northern Ireland, South Africa, Sweden, UK - Collaborations focused on a range of health improvement issues, such as HIV and disease prevention | - Interorganizational collaboration, defined as ‘partners engaging as a group to work synergistically across organizational boundaries toward a common intended goal’ |
| Sloper, P (2004). Facilitators and barriers for co-ordinated  multi-agency services^[[30]](#endnote-30)^ | - ‘Multi-agency’ working in public services - Wider context of national policies in UK to encouraging joint working between public sector agencies | - Multi-agency working not defined - Studies range in focused from ‘strategic level working’ (eg joint planning) to multi-disciplinary teams |
| Smith et al (2009). A systematic review of the impact of organizational partnerships on public health outcomes in England between 1997 and 2008^[[31]](#endnote-31)^ | - ‘Public health partnerships’ in England related to Health Action Zones, Health Improvement Programmes, and other national policy initiatives - Key partners include health care organizations, local government, housing, schools, and others | - Public health partnerships, defined as ‘organizational partnerships (of two or more organizational bodies), which aim to improve public health outcomes (through population health improvement and/or a reduction in health inequalities)’ |
| Whiteford et al (2014). System-level intersectoral linkages between the mental health and non-clinical support sectors: A qualitative systematic review^[[32]](#endnote-32)^ | - Collaboration between mental health and non-clinical services - Non-medical services included justice system, social services, education, vocational support, child welfare, substance abuse, employment, housing, government welfare, and other community-based services - Studies focused on people with mental health needs and homeless population | - ‘System-level intersectoral linkage’, defined as ‘any attempt to improve the service system for a defined population by implementing linkages between agencies and programs or reconfiguring or consolidating agencies at the policy, program or organisational level’ - Models of collaboration studied include: joint service planning and information exchange, multi-agency care plans, formal collaboration agreements (eg memoranda of understanding), staff training (including shared training), information sharing and joint information systems, joint service provision, co-location of services, single lead agency models |
| Wildridge et al (2004). How to create successful partnerships: a review of the literature^[[33]](#endnote-33)^ | - Context of government policy in UK promoting partnership working between health and social care agencies, as well as wider public services | - Not defined - Focus is largely on health and social care partnerships |
| Williams I (2009). Offender health and social care: a review of the evidence on inter-agency collaboration^[[34]](#endnote-34)^ | - Crime prevention and reduction partnerships in England - Partnerships associated with national policy initiatives to encourage collaboration between criminal justice agencies and health and social care - Agencies involved include criminal justice, health, local government, housing, and others | - Focuses on ‘formally instituted partnership bodies in England with a clear criminal justice remit’, including Drug (and Alcohol) Action Teams (D(A)ATs), Crime and Disorder Reduction Partnerships (CDRPs), Multi-Agency Public Protection-Arrangements (MAPPAs) and Youth Offending Teams (YOTs)’ |
| Winters et al (2016). Cross-sector provision in health and social care: an umbrella review^[[35]](#endnote-35)^ | - Partnerships between health care and social services - Studies focused on services for school-aged children, adults with comorbidity, adults living with a disability, veterans, nursing/care home patients, people living with HIV, primary care populations | - Cross-sector service provision. defined as ‘independent, yet interconnected sectors working together to better meet the needs of consumers and improve the quality and effectiveness of service provision' |
| Zakocs and Edwards (2006). What explains community coalition effectiveness? A review of the literature^[[36]](#endnote-36)^ | - Community coalitions in the US focused on population-level health improvement - Coalitions targeting US ‘neighborhoods, towns, cities, or counties’. Coalitions covering larger geographical areas (‘state, national, or international’) were excluded - Studies focused on coalitions targeting substance misuse, older people’s health, cancer, tobacco control, teen pregnancy, cardiovascular disease, alcohol use, and other health issues and risk factors | - Community coalitions, with coalitions defined as ‘inter-organizational, cooperative, and synergistic working alliances’ |

1. Anderson LM, Adeney KL, Shinn C, Safranek S, Buckner-Brown J, Krause LK. Community coalition‐driven interventions to reduce health disparities among racial and ethnic minority populations. Cochrane Database Syst Rev. 2015 Jun 15;(6):CD009905. [↑](#endnote-ref-1)
2. Andersson J, Ahgren B, Axelsson SB, Eriksson A, Axelsson R. Organizational approaches to collaboration in vocational rehabilitation-an international literature review. Int J Integr Care. 2011 Oct;11:e137. [↑](#endnote-ref-2)
3. Auschra C. Barriers to the integration of care in inter-organisational settings: a literature review. International Journal of Integrated Care, 2018;18(1)5:1–14. [↑](#endnote-ref-3)
4. Bagnall AM, Radley D, Jones R, Gately P, Nobles J, Van Dijk M, Blackshaw J, Montel S, Sahota P. Whole systems approaches to obesity and other complex public health challenges: a systematic review. BMC Public Health. 2019;19(1):8. [↑](#endnote-ref-4)
5. Baxter S, Johnson M, Chambers D, Sutton A, Goyder E, Booth A. The effects of integrated care: a systematic review of UK and international evidence. BMC Health Serv Res. 2018;18(1):350. [↑](#endnote-ref-5)
6. Cameron A, Lart R, Bostock L, Coomber C. Factors that promote and hinder joint and integrated working between health and social care services: a review of research literature. Health Soc Care Community. 2014 May;22(3):225-33. [↑](#endnote-ref-6)
7. Cooper M, Evens Y, Pybis J. Interagency collaboration in children and young people's mental health: a systematic review of outcomes, facilitating factors and inhibiting factors. Child Care Health Dev. 2016 May;42(3):325-42. [↑](#endnote-ref-7)
8. Corbin JH, Jones J, Barry MM. What makes intersectoral partnerships for health promotion work? A review of the international literature. Health Promotion International. 2016;33(1):4-26 [↑](#endnote-ref-8)
9. Davies SL, Goodman C, Bunn F, Victor C, Dickinson A, Iliffe S, Gage H, Martin W, Froggatt K. A systematic review of integrated working between care homes and health care services. BMC Health Serv Res. 2011;24(11):320. [↑](#endnote-ref-9)
10. Dowling B, Powell M, Glendinning C. Conceptualising successful partnerships. Health and Social Care in the Community. 2004;12(4):309-317. [↑](#endnote-ref-10)
11. Errecaborde KM et al. Factors that enable effective One Health collaborations - A scoping review of the literature. PLoS ONE [Electronic Resource] 14(12);2019. [↑](#endnote-ref-11)
12. Foster-Fishman PG, Berkowitz SL, Lounsbury DW, Jacobson S, Allen NA. Building collaborative capacity in community coalitions: a review and integrative framework. Am J Community Psychol. 2001;29(2):241-61. [↑](#endnote-ref-12)
13. Gannon-Leary P, Baines S, Wilson R. Collaboration and partnership: A review and reflections on a national project to join up local services in England. Journal of Interprofessional Care. 2006;20(6):665-674. [↑](#endnote-ref-13)
14. Green A et al. Cross-sector collaborations in Aboriginal and Torres Strait Islander childhood disability: A systematic integrative review and theory-based synthesis. International Journal for Equity in Health 13(1);2014. [↑](#endnote-ref-14)
15. Guglielmin, M., et al. A scoping review of the implementation of health in all policies at the local level. Health Policy 122(3): 284-292;2018. [↑](#endnote-ref-15)
16. Hayes SL, Mann MK, Morgan FM, Kelly MJ, Weightman AL. Collaboration between local health and local government agencies for health improvement. Cochrane Database of Systematic Reviews 2012, Issue 10. Art. No.: CD007825. DOI: 10.1002/14651858.CD007825.pub6. [↑](#endnote-ref-16)
17. Herdiana, H., et al. Intersectoral collaboration for the prevention and control of vector borne diseases to support the implementation of a global strategy: A systematic review. PLoS ONE [Electronic Resource] 13(10);2018. [↑](#endnote-ref-17)
18. Liljas AEM, Brattström F, Burström B, Schön P, Agerholm J, Impact of integrated care on patient-related outcomes among older people: a systematic review. International Journal of Integrated Care. 2019;19(3):1–16. [↑](#endnote-ref-18)
19. Lopez-Carmen V, McCalmana J, Benvenistea T, Askewb D, Spurlingb G, Langhama E, Bainbridgea R. Working together to improve the mental health of indigenous children: A systematic review. Child Youth Serv Rev. 2019;104:104408. [↑](#endnote-ref-19)
20. Mackie S, Darvill A. Factors enabling implementation of integrated health and social care: a systematic review. British Journal of Community Nursing. 2016;21(2):82-87. [↑](#endnote-ref-20)
21. Martin-Misener R, Valaitis R, Wong ST, Macdonald M, Meagher-Stewart D, Kaczorowski J, O-Mara L, Savage R, Austin P; Strengthening Primary Health Care through Public Health and Primary Care Collaborations Team. A scoping literature review of collaboration between primary care and public health. Prim Health Care Res Dev. 2012 Oct;13(4):327-46. [↑](#endnote-ref-21)
22. Mason A et al. Integrating funds for health and social care: an evidence review. Journal of health services research & policy 20(3): 177-188;2015. [↑](#endnote-ref-22)
23. Ndumbe-Eyoh S, Moffat H. Intersectoral action for health equity: a systematic review. BMC Public Health. 2013;13:1056. [↑](#endnote-ref-23)
24. Ogbonnaya IN, Keeney AJ. A systematic review of the effectiveness of interagency and cross-system collaborations in the United States to improve child welfare outcomes. Child Youth Serv Rev. 2018;94:225-245. [↑](#endnote-ref-24)
25. Perkins N, Smith K, Hunter DJ, Bambra C, Joyce K. ‘What counts is what works’? New Labour and partnerships in public health. Politics and Policy, 2010;38(1):101-117. [↑](#endnote-ref-25)
26. Rantala R, Bortz M, Armada F. Intersectoral action: local governments promoting health. Health Promotion International. 2014;29(Suppl 1):i92i102. [↑](#endnote-ref-26)
27. Roussos ST, Fawcett SB. A review of collaborative partnerships as a strategy for improving community health. Annu Rev Public Health. 2000;21:369-402. [↑](#endnote-ref-27)
28. Savic M, Best D, Manning V, Lubman D. Strategies to facilitate integrated care for people with alcohol and other drug problems: a systematic review. Subst Abuse Treat Prev Policy. 2017;12(1):19. [↑](#endnote-ref-28)
29. Seaton CL, Holm N, Bottorff JL, Jones-Bricker M, Errey S, Caperchione CM, Lamont S, Johnson ST, Healy T. Factors that impact the success of interorganizational health promotion collaborations: a scoping review. Am J Health Promot. 2018 May;32(4):1095-1109. [↑](#endnote-ref-29)
30. Sloper, P. Facilitators and barriers for co-ordinated multi-agency services. Child: Care. Health and Development. 2004; 30(6): 571–80. [↑](#endnote-ref-30)
31. Smith KE, Bambra C, KE Joyce, N Perkins, DJ Hunter, Blenkinsopp EA. Partners in health? A systematic review of the impact of organizational partnerships on public health outcomes in England between 1997 and 2008. Journal of Public Health. 2009;31(2):210-221. [↑](#endnote-ref-31)
32. Whiteford, H., et al. System-level intersectoral linkages between the mental health and non-clinical support sectors: A qualitative systematic review. Australian and New Zealand Journal of Psychiatry 48(10):895-906;2014. [↑](#endnote-ref-32)
33. Wildridge V, Childs S, Cawthra L, Madge B. How to create successful partnerships: a review of the literature. Health Information and Libraries Journal. 2004;21:3–19. [↑](#endnote-ref-33)
34. Williams I. Offender health and social care: a review of the evidence on inter-agency collaboration. Health and Social Care in the Community. 2009;17(6):573–580. [↑](#endnote-ref-34)
35. Winters S, Magalhaes L, Kinsella EA, Kothari A. Cross-sector provision in health and social care: an umbrella review. Int J Integr Care. 2016;16(1):1-19. [↑](#endnote-ref-35)
36. Zakocs RC, Edwards EM. What explains community coalition effectiveness? A review of the literature. Am J Prev Med. 30(4):351-61;2006. [↑](#endnote-ref-36)
