# Supplementary material for: The impacts of collaboration between local health care and non-health care organizations and factors shaping how they work: a systematic review of reviews
Source: BMC Public Health. 2021 Apr 19;21:753. doi: 10.1186/s12889-021-10630-1 (PMC8054696; doi:10.1186/s12889-021-10630-1)
Supplement: Supplementary file 3 — Additional file 3: Table S2. Study quality assessments [file 12889_2021_10630_MOESM3_ESM.docx]

**TABLE S2: study quality assessments**

| **Study** | **Study design** | **AMSTAR 2 assessment for reviews reporting evidence on collaboration outcomes^[[1]](#footnote-1)^** | **CASP checklist for studies only reporting evidence on factors influencing collaboration^[[2]](#footnote-2)^** | **How authors assessed risk of bias for studies included in their review** |
| --- | --- | --- | --- | --- |
| Anderson et al (2015). Community coalition‐driven interventions to reduce health disparities among racial and ethnic minority populations^[[3]](#endnote-1)^ | - Systematic review - 58 studies included | High | NA | - Assessed using Cochrane risk of bias tool for RCTs and Effective Practice and Organization of Care (EPOC) risk of bias tool for other studies - Overall, studies included showed ‘moderate to high risk of bias’, particularly in relation to selection bias |
| Andersson et al (2011). Organizational approaches to collaboration in vocational rehabilitation-an international literature review^[[4]](#endnote-2)^ | - Review - 62 studies included | NA | 1,2 | - No formal quality assessment |
| Auschra C (2018). Barriers to the integration of care in inter-organisational settings:  a literature review^[[5]](#endnote-3)^ | - Systematic review - 40 studies included | NA | 1,5 | - No formal quality assessment |
| Bagnall et al (2019). Whole systems approaches to obesity and other complex public health challenges: a systematic review^[[6]](#endnote-4)^ | - Systematic review - 35 studies included | Low | NA | - Assessed using checklists adapted from the National Institute for Health and Care Excellence Public Health methods guidance, and the Critical Skills Appraisal Programme (CASP) |
| Baxter et al (2018). The effects of integrated care: a systematic review of UK and international evidence^[[7]](#endnote-5)^ | - Systematic review - 167 studies included | High | NA | - Quality assessment using a variety of checklists depending on study type, including Cochrane criteria and National Institutes of Health checklists - Evidence assessments for each outcome category (see table 3) |
| Cameron et al (2014). Factors that promote and hinder joint and integrated working between health and social care services: a review of research literature^[[8]](#endnote-6)^ | - Review - 46 papers, reporting on 30 studies | Critically low | NA | - No formal quality assessment - Authors note that the evidence had several limitations, including small scale studies and few with comparative design |
| Cooper et al (2016). Interagency collaboration in children and young people's mental health: a systematic review of outcomes, facilitating factors and inhibiting factors^[[9]](#endnote-7)^ | - Systematic review - 33 studies included | Critically low | NA | - Assessed using CASP checklists - Quantitative studies: assessed as being ‘suitable’ for the investigations conducted—though studies used correlational designs and assessment of outcomes at follow-up was limited - Qualitative studies: 10 assessed as ‘valuable’, 10 ‘fairly valuable’, one ‘not valuable’ |
| Corbin (2016). What makes intersectoral partnerships for health promotion work? A review of the international literature^[[10]](#endnote-8)^ | - Review - 26 studies included | NA | 1,2 | - No formal quality assessment used - Authors note that few studies comprehensively assess partnership processes, factors, or their interaction |
| Davies et al (2011). A systematic review of integrated working between care homes and health care services^[[11]](#endnote-9)^ | - Systematic review - 17 studies included | Critically low | NA | - Assessed using checklists based on the Cochrane Collaboration risk of bias tool and Spencer et al’s quality assessment checklist for qualitative studies |
| Dowling et al (2004). Conceptualising successful partnerships^[[12]](#endnote-10)^ | - Review - 36 studies included | Critically low | NA | - No formal quality assessment - Authors describe weaknesses in evidence |
| Errecaborde et al (2019). Factors that enable effective one health collaborations: a scoping review of the literature^[[13]](#endnote-11)^ | - Review - 50 studies included | Critically low | NA | - No formal quality assessment |
| Foster-Fishman et al (2001). Building collaborative capacity in community coalitions: a review and integrative framework^[[14]](#endnote-12)^ | - Review - 80 studies included | NA | - | - No formal quality assessment |
| Gannon-Leary et al (2006). Collaboration and partnership: A review and reflections on a national project to join up local services in England^[[15]](#endnote-13)^ | - Narrative review - Studies included not defined | NA | - | - No formal quality assessment |
| Green et al (2014). Cross-sector collaborations in Aboriginal and Torres Strait Islander childhood disability: A systematic integrative review and theory-based synthesis^[[16]](#endnote-14)^ | - Systematic review - 31 studies included | NA | 1,2,3,4 | - Assessed using multiple checklists depending on study design, including Kitto et al’s quality assessment tool for qualitative studies, the STROBE checklist for observational studies, AMSTAR for review articles, the MMAT for mixed methods studies, and the TREND checklist for non-randomized intervention studies |
| Guglielmin et al (2018). A scoping review of the implementation of health in all policies at the local level^[[17]](#endnote-15)^ | - Review - 27 studies included | NA | 1.5 | - No formal quality assessment |
| Hayes et al (2012). Collaboration between local health and local government agencies for health improvement^[[18]](#endnote-16)^ | - Systematic review and meta-analysis - 16 studies included - 11 studies used for meta-analysis | High | NA | - Assessed using EPOC data collection checklist - RCTs: one low risk of bias, one medium of risk of bias, two high risk of bias - Non-randomized studies: one medium risk of bias, others high risk of bias |
| Herdiana et al (2018). Intersectoral collaboration for the prevention and control of vector borne diseases to support the implementation of a global strategy: a systematic review^[[19]](#endnote-17)^ | - Systematic review - 50 articles included | Low | NA | - Assessed using Cochrane handbook - Quantitative studies: 10 rated ‘strong’, 9 ‘moderate’, 31 ‘poor’ - Qualitative studies: not reported |
| Liljas et al (2019). Impact of integrated care on patient-related outcomes among older people: a systematic review^[[20]](#endnote-18)^ | - Systematic review - 12 studies included | Low | NA | - Assessed using checklists developed by the Swedish Agency for Health Technology Assessment and Assessment of Social Services - Six studies low risk of bias, five studies moderate risk of bias |
| Lopez-Carmen et al (2019). Working together to improve the mental health of indigenous children: A systematic review^[[21]](#endnote-19)^ | - Systematic review - 11 studies included | Critically low | NA | - No formal quality assessment (though quality ratings are described in discussion) - Authors note that most studies were descriptive accounts of service integration, with few impact evaluations |
| Mackie and Darvill (2016). Factors enabling implementation of integrated health and social care: a systematic review^[[22]](#endnote-20)^ | - Systematic review - 7 studies included | NA | 1,4,5 | - Assessed using CASP checklist for systematic reviews - Overall, quality of studies assessed as low |
| Martin-Misener et al (2012). Strengthening Primary Health Care through Public Health and Primary Care Collaborations Team. A scoping literature review of collaboration between primary care and public health^[[23]](#endnote-21)^ | - Review - 114 studies included | Critically low | NA | - No formal quality assessment - Authors note that a large proportion of the articles were descriptive accounts of collaboration, and 75% used qualitative, mixed methods, or cross-sectional design |
| Mason et al (2015). Integrating funds for health and social care: an evidence review^[[24]](#endnote-22)^ | - Review - 122 studies included, reporting on 38 initiatives | Critically low | NA | - No formal quality assessment |
| Ndumbe-Eyoh and Moffat (2013). Intersectoral action for health equity: a systematic review^[[25]](#endnote-23)^ | - Systematic review - 17 articles included | Low | NA | - Assessed using three different tools (for systematic reviews, qualitative and quantitative studies) - Systematic review: strong - Quantitative studies: one strong, five moderate, eight weak - Qualitative studies: no overall rating |
| Ogbonnaya and Keeney (2018). A systematic review of the effectiveness of interagency and cross-system  collaborations in the United States to improve child welfare outcomes^[[26]](#endnote-24)^ | - Systematic review and meta-analysis - 11 studies included | Critically low | NA | - Assessed using National Institute for Health (NIH)/National Heart, Lung, and Blood Institute (NHLBI) tools—one assessment for experimental studies and one for quasi-experimental studies - Narrative overview of study quality: study quality varied, with limited information to assess experimental studies |
| Perkins et al (2010). ‘What counts is what works’? New Labour and partnerships in public health^[[27]](#endnote-25)^ | - Systematic review - 31 studies included | NA | 1,2,4,5 | - See Smith et al (2009) |
| Rantala et al (2014). Intersectoral action: local governments promoting health^[[28]](#endnote-26)^ | - Review - Studies included not defined (but 25 case studies identified) | NA | 1,5 | - No formal quality assessment |
| Roussos and Fawcett (2000). A review of collaborative partnerships as a strategy for improving community health^[[29]](#endnote-27)^ | - Review - 34 studies included, reporting on 252 partnerships | Critically low | NA | - No formal quality assessment - Authors describe several limitations of the evidence (‘weak outcomes, contradictory results, or null effects were found in the more methodologically rigorous studies’) |
| Savic et al (2017). Strategies to facilitate integrated care for people with alcohol and other drug problems: a systematic review^[[30]](#endnote-28)^ | - Systematic review - 14 studies included | NA | 1,2 | - No formal quality assessment |
| Seaton et al (2018). Factors that impact the success of interorganizational health promotion collaborations: a scoping review^[[31]](#endnote-29)^ | - Systematic review - 25 studies included | NA | 1,2,4,5 | - Assessed using tool adapted from Harden et al. |
| Sloper, P (2004). Facilitators and barriers for co-ordinated  multi-agency services^[[32]](#endnote-30)^ | - Review - Studies included not defined | Critically low | NA | - No formal quality assessment |
| Smith et al (2009). A systematic review of the impact of organizational partnerships on public health outcomes in England between 1997 and 2008^[[33]](#endnote-31)^ | - Systematic review - 15 studies included | Low | NA | - Assessed against critical appraisal criteria, adapted from two instruments - Authors note that the evidence had several limitations, such as short follow-up and potential contamination between control and intervention groups |
| Whiteford et al (2014). System-level intersectoral linkages between the mental health and non-clinical support sectors: A qualitative systematic review^[[34]](#endnote-32)^ | - Systematic review - 40 studies included | Critically low | NA | - Assessed using National Health and Medical Research Councils (Australia) - Studies assessed from level 1 (highest quality) to level 4 (lowest quality) - 10 studies level 2, 14 studies level 3, 16 studies level 4 |
| Wildridge et al (2004). How to create successful partnerships: a review of the literature^[[35]](#endnote-33)^ | - Review - Studies included not defined | NA | - | - No formal quality assessment |
| Williams I (2009). Offender health and social care: a review of the evidence on inter-agency collaboration^[[36]](#endnote-34)^ | - Narrative review - Studies included not defined | NA | 1 | - No formal quality assessment |
| Winters et al (2016). Cross-sector provision in health and social care: an umbrella review^[[37]](#endnote-35)^ | - Umbrella review - 16 studies included | Critically low | NA | - Assessed using Joanna Briggs Critical Appraisal Checklist (but several low rated articles were included due to relevance) |
| Zakocs and Edwards (2006). What explains community coalition effectiveness? A review of the literature^[[38]](#endnote-36)^ | - Review - 26 articles included | NA | 1,2,5 | - No formal quality assessment |

1. We assessed studies against the 16 items in the AMSTAR 2 instrument. Item 2—having a protocol registered before commencement of the review—was not deemed a critical domain when constructing the overall ratings, given that papers were included from a wide range of disciplines where this would not necessarily be expected. [↑](#footnote-ref-1)
2. We assessed studies against the first 5 items in the CASP instrument. The five items are: (1) Did the review have a clearly focused question? (2) Did the authors look for the right kind of papers? (3) Do you think all the important, relevant studies were included? (4) Did the review’s authors do enough to assess quality of the included studies? (5) If the results of the review have been combined, was it reasonable to do so? For item 5, we scored papers as meeting this criterion if the findings of individual studies were clearly displayed or described, or if the paper clearly illustrated the presence or absence of review findings or themes between the studies included. Given there is no overall rating in the CASP instrument, we include the number 1-5 for each paper only if it was deemed to fully meet the corresponding criterion. [↑](#footnote-ref-2)
3. Anderson LM, Adeney KL, Shinn C, Safranek S, Buckner-Brown J, Krause LK. Community coalition‐driven interventions to reduce health disparities among racial and ethnic minority populations. Cochrane Database Syst Rev. 2015 Jun 15;(6):CD009905. [↑](#endnote-ref-1)
4. Andersson J, Ahgren B, Axelsson SB, Eriksson A, Axelsson R. Organizational approaches to collaboration in vocational rehabilitation-an international literature review. Int J Integr Care. 2011 Oct;11:e137. [↑](#endnote-ref-2)
5. Auschra C. Barriers to the integration of care in inter-organisational settings: a literature review. International Journal of Integrated Care, 2018;18(1)5:1–14. [↑](#endnote-ref-3)
6. Bagnall AM, Radley D, Jones R, Gately P, Nobles J, Van Dijk M, Blackshaw J, Montel S, Sahota P. Whole systems approaches to obesity and other complex public health challenges: a systematic review. BMC Public Health. 2019;19(1):8. [↑](#endnote-ref-4)
7. Baxter S, Johnson M, Chambers D, Sutton A, Goyder E, Booth A. The effects of integrated care: a systematic review of UK and international evidence. BMC Health Serv Res. 2018;18(1):350. [↑](#endnote-ref-5)
8. Cameron A, Lart R, Bostock L, Coomber C. Factors that promote and hinder joint and integrated working between health and social care services: a review of research literature. Health Soc Care Community. 2014 May;22(3):225-33. [↑](#endnote-ref-6)
9. Cooper M, Evens Y, Pybis J. Interagency collaboration in children and young people's mental health: a systematic review of outcomes, facilitating factors and inhibiting factors. Child Care Health Dev. 2016 May;42(3):325-42. [↑](#endnote-ref-7)
10. Corbin JH, Jones J, Barry MM. What makes intersectoral partnerships for health promotion work? A review of the international literature. Health Promotion International. 2016;33(1):4-26 [↑](#endnote-ref-8)
11. Davies SL, Goodman C, Bunn F, Victor C, Dickinson A, Iliffe S, Gage H, Martin W, Froggatt K. A systematic review of integrated working between care homes and health care services. BMC Health Serv Res. 2011;24(11):320. [↑](#endnote-ref-9)
12. Dowling B, Powell M, Glendinning C. Conceptualising successful partnerships. Health and Social Care in the Community. 2004;12(4):309-317. [↑](#endnote-ref-10)
13. Errecaborde KM et al. Factors that enable effective One Health collaborations - A scoping review of the literature. PLoS ONE [Electronic Resource] 14(12);2019. [↑](#endnote-ref-11)
14. Foster-Fishman PG, Berkowitz SL, Lounsbury DW, Jacobson S, Allen NA. Building collaborative capacity in community coalitions: a review and integrative framework. Am J Community Psychol. 2001;29(2):241-61. [↑](#endnote-ref-12)
15. Gannon-Leary P, Baines S, Wilson R. Collaboration and partnership: A review and reflections on a national project to join up local services in England. Journal of Interprofessional Care. 2006;20(6):665-674. [↑](#endnote-ref-13)
16. Green A et al. Cross-sector collaborations in Aboriginal and Torres Strait Islander childhood disability: A systematic integrative review and theory-based synthesis. International Journal for Equity in Health 13(1);2014. [↑](#endnote-ref-14)
17. Guglielmin, M., et al. A scoping review of the implementation of health in all policies at the local level. Health Policy 122(3): 284-292;2018. [↑](#endnote-ref-15)
18. Hayes SL, Mann MK, Morgan FM, Kelly MJ, Weightman AL. Collaboration between local health and local government agencies for health improvement. Cochrane Database of Systematic Reviews 2012, Issue 10. Art. No.: CD007825. DOI: 10.1002/14651858.CD007825.pub6. [↑](#endnote-ref-16)
19. Herdiana, H., et al. Intersectoral collaboration for the prevention and control of vector borne diseases to support the implementation of a global strategy: A systematic review. PLoS ONE [Electronic Resource] 13(10);2018. [↑](#endnote-ref-17)
20. Liljas AEM, Brattström F, Burström B, Schön P, Agerholm J, Impact of integrated care on patient-related outcomes among older people: a systematic review. International Journal of Integrated Care. 2019;19(3):1–16. [↑](#endnote-ref-18)
21. Lopez-Carmen V, McCalmana J, Benvenistea T, Askewb D, Spurlingb G, Langhama E, Bainbridgea R. Working together to improve the mental health of indigenous children: A systematic review. Child Youth Serv Rev. 2019;104:104408. [↑](#endnote-ref-19)
22. Mackie S, Darvill A. Factors enabling implementation of integrated health and social care: a systematic review. British Journal of Community Nursing. 2016;21(2):82-87. [↑](#endnote-ref-20)
23. Martin-Misener R, Valaitis R, Wong ST, Macdonald M, Meagher-Stewart D, Kaczorowski J, O-Mara L, Savage R, Austin P; Strengthening Primary Health Care through Public Health and Primary Care Collaborations Team. A scoping literature review of collaboration between primary care and public health. Prim Health Care Res Dev. 2012 Oct;13(4):327-46. [↑](#endnote-ref-21)
24. Mason A et al. Integrating funds for health and social care: an evidence review. Journal of health services research & policy 20(3): 177-188;2015. [↑](#endnote-ref-22)
25. Ndumbe-Eyoh S, Moffat H. Intersectoral action for health equity: a systematic review. BMC Public Health. 2013;13:1056. [↑](#endnote-ref-23)
26. Ogbonnaya IN, Keeney AJ. A systematic review of the effectiveness of interagency and cross-system collaborations in the United States to improve child welfare outcomes. Child Youth Serv Rev. 2018;94:225-245. [↑](#endnote-ref-24)
27. Perkins N, Smith K, Hunter DJ, Bambra C, Joyce K. ‘What counts is what works’? New Labour and partnerships in public health. Politics and Policy, 2010;38(1):101-117. [↑](#endnote-ref-25)
28. Rantala R, Bortz M, Armada F. Intersectoral action: local governments promoting health. Health Promotion International. 2014;29(Suppl 1):i92i102. [↑](#endnote-ref-26)
29. Roussos ST, Fawcett SB. A review of collaborative partnerships as a strategy for improving community health. Annu Rev Public Health. 2000;21:369-402. [↑](#endnote-ref-27)
30. Savic M, Best D, Manning V, Lubman D. Strategies to facilitate integrated care for people with alcohol and other drug problems: a systematic review. Subst Abuse Treat Prev Policy. 2017;12(1):19. [↑](#endnote-ref-28)
31. Seaton CL, Holm N, Bottorff JL, Jones-Bricker M, Errey S, Caperchione CM, Lamont S, Johnson ST, Healy T. Factors that impact the success of interorganizational health promotion collaborations: a scoping review. Am J Health Promot. 2018 May;32(4):1095-1109. [↑](#endnote-ref-29)
32. Sloper, P. Facilitators and barriers for co-ordinated multi-agency services. Child: Care. Health and Development. 2004; 30(6): 571–80. [↑](#endnote-ref-30)
33. Smith KE, Bambra C, KE Joyce, N Perkins, DJ Hunter, Blenkinsopp EA. Partners in health? A systematic review of the impact of organizational partnerships on public health outcomes in England between 1997 and 2008. Journal of Public Health. 2009;31(2):210-221. [↑](#endnote-ref-31)
34. Whiteford, H., et al. System-level intersectoral linkages between the mental health and non-clinical support sectors: A qualitative systematic review. Australian and New Zealand Journal of Psychiatry 48(10):895-906;2014. [↑](#endnote-ref-32)
35. Wildridge V, Childs S, Cawthra L, Madge B. How to create successful partnerships: a review of the literature. Health Information and Libraries Journal. 2004;21:3–19. [↑](#endnote-ref-33)
36. Williams I. Offender health and social care: a review of the evidence on inter-agency collaboration. Health and Social Care in the Community. 2009;17(6):573–580. [↑](#endnote-ref-34)
37. Winters S, Magalhaes L, Kinsella EA, Kothari A. Cross-sector provision in health and social care: an umbrella review. Int J Integr Care. 2016;16(1):1-19. [↑](#endnote-ref-35)
38. Zakocs RC, Edwards EM. What explains community coalition effectiveness? A review of the literature. Am J Prev Med. 30(4):351-61;2006. [↑](#endnote-ref-36)
