# Supplementary material for: The impacts of collaboration between local health care and non-health care organizations and factors shaping how they work: a systematic review of reviews
Source: BMC Public Health. 2021 Apr 19;21:753. doi: 10.1186/s12889-021-10630-1 (PMC8054696; doi:10.1186/s12889-021-10630-1)
Supplement: Supplementary file 4 — Additional file 4: Table S3. Summary of evidence on collaboration impacts [file 12889_2021_10630_MOESM4_ESM.docx]

**TABLE S3: summary of evidence on collaboration impacts**

| **Collaboration impacts** | | | |  |  |
| --- | --- | --- | --- | --- | --- |
| **Paper** | **Outcomes studied** | *Health outcomes* | *Service use and quality* | *Resource use and spending* | *Process impacts* |
| Hayes et al (2012). Collaboration between local health and local government agencies for health improvement^[[1]](#endnote-1)^ * | - Measures of improved health, health status, survival, or health-related lifestyle factors - Studies included with any measure of mortality, morbidity, or behaviour change | - Overall, little or no reliable evidence of health benefits - Meta-analysis of three studies investigating impact on mortality found no effect (pooled relative risk = 1.04 in favour of control, 95% CI 0.92 to 1.17) and no heterogeneity (I^2^ = 0%) - Meta-analysis of five studies investigating impact on mental health found a small effect favouring the intervention (standardized mean difference = -0.28, 95% CI -0.52 to -0.04) with evidence of heterogeneity (I^2^ = 87%) - Meta-analysis of two studies investigating impact on function found a small improvement in the global assessment of function symptoms score scale (pooled mean difference [on a scale of 1-100] = -2.63, 95% CI -5.16 to -0.10) and no heterogeneity (I^2^ = 0%) - Meta-analysis of five studies investigating impact on physical health found no evidence of improved physical health (standardized mean difference = -0.01, 95% CI -0.10 to 0.07) and little evidence of heterogeneity (I^2^ = 16%) - Meta-analysis of three studies investigating impact on quality of life found no significant difference in quality of life (standardized mean difference = -0.08, 95% CI -0.44 to 0.27) with evidence of heterogeneity (I^2^ = 83%) | NA | - Some studies reported additional costs associated with partnership interventions | NA |
| Baxter et al (2018). The effects of integrated care: a systematic review of UK and international evidence^[[2]](#endnote-2)^ * | - Service delivery outcomes, including effectiveness, efficiency, or quality, and/or the effect on patients and staff | NA | - Stronger evidence for improvements in patient satisfaction, improvements in perceived quality of care, and improvements in access to some services - Inconsistent evidence related to number of clinician contacts, number of GP appointments, length of stay, unscheduled admissions, number of admissions, re-admissions, attendance at accident and emergency, quality of care standards, staff work experience, community care activity, secondary care activity, overall healthcare utilization - Limited evidence on prescribing rates, access to resources, time spent in accident and emergency department, number of incidents/complaints, identification of unmet need | - Inconsistent evidence related to cost of provision | NA |
| Anderson et al (2015). Community coalition‐driven interventions to reduce health disparities among racial and ethnic minority populations^[[3]](#endnote-3)^ * | - Measures of mortality (eg all-cause death within period of study), morbidity (eg quality of life), and health-behaviors (eg smoking and alcohol consumption) - Also focused on costs of interventions | - Broad‐scale community system level change strategies led to little or no difference in measures of health behavior or health status - Broad health and social care system level strategies led to small beneficial changes in measures of health behavior or health status in large samples of community residents - Lay community health outreach worker interventions led to beneficial changes in health behavior measures of moderate magnitude in large samples of community residents - Lay community health outreach worker interventions may lead to beneficial changes in health status measures in large samples of community residents; however, results were not consistent across studies - Group‐based health education led by professional staff resulted in moderate improvement in measures of health behavior - Adverse outcomes of community coalition‐led interventions not reported - Moderate to substantial heterogeneity (I^2^ > 50%). in effects across studies | NA | - Financial data on interventions not reported | NA |
| Smith et al (2009). A systematic review of the impact of organizational partnerships on public health outcomes in England between 1997 and 2008^[[4]](#endnote-4)^ ‡ | - Public health outcomes, defined as ‘health improvement and/or a reduction in health inequalities’ - Impact could be direct (eg by improving self-reported health) or indirect (eg by raising profile of health inequalities) | - Quantitative studies found no intervention effect (two studies found no improvements compared to other areas) or mixed effects (one study found improvements on some indicators but worse performance on others) on health outcomes, such as morbidity and mortality - One mixed methods study found that people had been supported to adopt healthier lifestyles | NA | NA | - Qualitative studies found that partnership initiatives had helped embed or increase focus on health inequalities |
| Liljas et al (2019). Impact of integrated care on patient-related outcomes among older people: a systematic review^[[5]](#endnote-5)^ ‡ | - Patient satisfaction, hospital admission, length of hospital stay, hospital readmission, mortality | - No studies examining mortality reported significant changes in mortality rates | - Mixed impacts on hospital admissions, readmissions, and length of stay - Mixed impacts on patient satisfaction | NA | NA |
| Ndumbe-Eyoh and Moffat (2013). Intersectoral action for health equity: a systematic review^[[6]](#endnote-6)^ ‡ | - Impact on health equity or social determinants of health, such as housing or employment, for deprived groups | - Mixed impacts on health outcomes - Limited evidence on equity impacts | - More downstream interventions ‘moderately effective’ in increasing access to services for marginalized groups | NA | NA |
| Bagnall et al (2019). Whole systems approaches to obesity and other complex public health challenges: a systematic review^[[7]](#endnote-7)^ ‡ | - Focused broadly on evidence of effectiveness and cost-effectiveness of whole systems approaches | - Most studies reported some positive effects, including on health behaviors and BMI - Some studies reported positive effects on wider public health outcomes, including on smoking rates, exercise, and diet | NA | - Limited evidence on cost-effectiveness | NA |
| Herdiana et al (2018). Intersectoral collaboration for the prevention and control of vector borne diseases to support the implementation of a global strategy: a systematic review^[[8]](#endnote-8)^ ‡ | - Outcomes related to the prevention and control of vector borne diseases, such as disease incidence or prevalence | - Most studies measuring disease indicators reported positive effects, such as reduction of cases | - Most studies measuring vector variables (adult density, pupae or larval indices) reported declining vector indices - Improvements in access to intervention and treatment | NA | NA |
| Davies et al (2011). A systematic review of integrated working between care homes and health care services^[[9]](#endnote-9)^ § | - Health and wellbeing (eg health status, quality of life), service use (eg hospital admissions), cost savings, process-related outcomes (eg quality of care and staff satisfaction) | - Some improvements in outcomes reported, but the majority of studies found that the intervention had mixed effects or no effect | - Some improvements in outcomes reported, but the majority of studies found that the intervention had mixed effects or no effect | - Insufficient evidence on costs | NA |
| Mason et al (2015). Integrating funds for health and social care: an evidence review^[[10]](#endnote-10)^ § | - Focused broadly on effectiveness or cost-effectiveness, including a range of health and service level outcomes | - Most studies assessing health outcomes (including health-related quality of life, physical functioning, depression and anxiety, mortality, carer burden) found no significant difference from usual care. Findings from other studies were mixed | - Impact on secondary care utilization was mixed - Impact on quality of care and user experience was mixed | - Impact on secondary care costs was mixed | - Some studies found unintended consequences, such as ‘upcoding’ |
| Ogbonnaya and Keeney (2018). A systematic review of the effectiveness of interagency and cross-system  collaborations in the United States to improve child welfare outcomes^[[11]](#endnote-11)^ § | - Outcomes related to ‘safety, permanency, and well-being for child welfare involved families’ | NA | - Family drug treatment court (FDTC) collaboration intervention positively associated with treatment entry (odds ratio [OR] = 2.94, 95% CI 1.50 to 5.75, with evidence of heterogeneity I^2^ = 86%) and completion (OR = 2.07, 95% CI 1.26 to 3.41, with evidence of heterogeneity I^2^ = 84%) of substance use services - FDTC (OR = 2.40, 95% CI 1.75 to 3.29, with evidence of heterogeneity I^2^ = 71%) and recovery coaches (OR = 1.52, 95% CI 1.17 to 1.99, with little evidence of heterogeneity I^2^ = 30%) were positively associated with likelihood of reunification - Relationship between FDTC and days to reunification less positive across studies (standardized mean difference = 0.47, 95% CI 0.25, 0.69, with some evidence of heterogeneity I^2^ = 48%). | NA | NA |
| Lopez-Carmen et al (2019) Working together to improve the mental health of indigenous children: A systematic review^[[12]](#endnote-12)^ § | - Effects of integrated mental health services for indigenous children, including children’s mental health outcomes | - Improvements in children’s psychosocial functioning, stress management, and individual ‘empowerment’ - ‘Empowerment’ of families and communities | - Improved access to services, utilization | NA | - Greater collaboration between health and non-health services and ‘strengthened organizational capacity’ |
| Whiteford et al (2014). System-level intersectoral linkages between the mental health and non-clinical support sectors: A qualitative systematic review^[[13]](#endnote-13)^ § | - Focused broadly on outcomes for services or clients from intersectoral linkages between clinical and non-clinical mental health services | NA | - Improved accommodation stability, reduced child foster placements, reduced recidivism and involvement with the juvenile justice system, improved employment related outcomes - Studies of one program to address homelessness among people with severe mental illness did not lead to improved outcomes | - Improved efficiency, though also examples of cost shifting | - Improvements in interagency communication, mutual understanding of services |
| Martin-Misener et al (2012). Strengthening Primary Health Care through Public Health and Primary Care Collaborations Team. A scoping literature review of collaboration between primary care and public health^[[14]](#endnote-14)^ § | - Focused broadly on outcomes of primary care and public health collaborations, including outcomes related to individuals and populations, health professionals, and health service delivery | - Improvements in chronic disease management, disease control, maternal and child health | - Improvements in access to care, immunization rates, and care processes, such as needs assessments | NA | - Financial incentives for health promotion may skew priorities away from efforts to reduce health inequities - Concerns among primary care staff about reduced time for medical care |
| Cameron et al (2014). Factors that promote and hinder joint and integrated working between health and social care services: a review of research literature^[[15]](#endnote-15)^ § | - Focused broadly on evidence of ‘effectiveness’ | - Some studies reported improvements in quality of life, but no or marginal improvements in studies with comparative designs | - Some studies of particular service models (eg intermediate care) found potential reductions in inappropriate admissions to institutional settings | - Most studies did not find cost savings | NA |
| Winters et al (2016). Cross-sector provision in health and social care: an umbrella review^[[16]](#endnote-16)^ § | - Focused broadly on ‘impacts related to cross-sector service provision and service delivery’ | - Majority of studies did not report positive impacts on outcomes (outcomes were weakly defined) | - Majority of studies did not report positive impacts on outcomes (outcomes were weakly defined) - Four reviews reported positive impacts, including improvements in access and potential reductions in length of stay and readmissions to institutional settings | NA | NA |
| Roussos and Fawcett (2000). A review of collaborative partnerships as a strategy for improving community health^[[17]](#endnote-17)^ § | - Population-level health outcomes and behaviors (eg smoking or physical activity) | - Insufficient evidence related to population level outcomes - Collaborative practice can contribute to change in community health behaviors | NA | NA | NA |
| Cooper et al (2016). Interagency collaboration in children and young people's mental health: a systematic review of outcomes, facilitating factors and inhibiting factors^[[18]](#endnote-18)^ § | - Focused broadly on outcomes of interagency collaboration across children and young people’s mental health services, such as health status and service use | - One study found a positive association between collaboration and mental health status | - Mixed impacts, including on access and quality of services (with some studies reporting positive impacts and access and its equitable provision, but others reporting negative impacts on access and quality) - Collaboration generally viewed positively by staff, patients, carers | NA | - Five studies reported positive attitudes to collaboration from staff, but one study found that staff reported increased time burden, management difficulties, challenges to professional identities, and other issues |
| Sloper, P (2004). Facilitators and barriers for co-ordinated  multi-agency services^[[19]](#endnote-19)^ § | - Focused broadly on ‘outcomes for service users’, such as quality of life and service use | - Limited evidence | - Limited evidence | NA | NA |
| Errecaborde et al (2019). Factors that enable effective one health collaborations: a scoping review of the literature^[[20]](#endnote-20)^ § | - Focused broadly on the outcomes and effectiveness of collaborations around health events | - Vast majority of studies did not report on outcomes - Impacts reported include decreased mortality (one study), reduction in MRSA cases (one study), improved safety | NA | - Vast majority of studies did not report on outcomes - One study reported reductions in cost | - Process impacts reported were increased stakeholder buy-in and professional development opportunities |
| Dowling et al (2004). Conceptualising successful partnerships^[[21]](#endnote-21)^ § | - Impacts related to ‘service provision to users and carers, or to the wider interface of health and social care’ | - No clear or consistent evidence of improvements | - No clear or consistent evidence of improvements | NA | NA |
| *AMSTAR 2 overall confidence assessment:*  * High  † Medium  ‡ Low  § Critically low | | | | | |

1. Hayes SL, Mann MK, Morgan FM, Kelly MJ, Weightman AL. Collaboration between local health and local government agencies for health improvement. Cochrane Database of Systematic Reviews 2012, Issue 10. Art. No.: CD007825. DOI: 10.1002/14651858.CD007825.pub6. [↑](#endnote-ref-1)
2. Baxter S, Johnson M, Chambers D, Sutton A, Goyder E, Booth A. The effects of integrated care: a systematic review of UK and international evidence. BMC Health Serv Res. 2018;18(1):350. [↑](#endnote-ref-2)
3. Anderson LM, Adeney KL, Shinn C, Safranek S, Buckner-Brown J, Krause LK. Community coalition‐driven interventions to reduce health disparities among racial and ethnic minority populations. Cochrane Database Syst Rev. 2015 Jun 15;(6):CD009905. [↑](#endnote-ref-3)
4. Smith KE, Bambra C, KE Joyce, N Perkins, DJ Hunter, Blenkinsopp EA. Partners in health? A systematic review of the impact of organizational partnerships on public health outcomes in England between 1997 and 2008. Journal of Public Health. 2009;31(2):210-221. [↑](#endnote-ref-4)
5. Liljas AEM, Brattström F, Burström B, Schön P, Agerholm J, Impact of integrated care on patient-related outcomes among older people: a systematic review. International Journal of Integrated Care. 2019;19(3):1–16. [↑](#endnote-ref-5)
6. Ndumbe-Eyoh S, Moffat H. Intersectoral action for health equity: a systematic review. BMC Public Health. 2013;13:1056. [↑](#endnote-ref-6)
7. Bagnall AM, Radley D, Jones R, Gately P, Nobles J, Van Dijk M, Blackshaw J, Montel S, Sahota P. Whole systems approaches to obesity and other complex public health challenges: a systematic review. BMC Public Health. 2019;19(1):8. [↑](#endnote-ref-7)
8. Herdiana, H., et al. Intersectoral collaboration for the prevention and control of vector borne diseases to support the implementation of a global strategy: A systematic review. PLoS ONE [Electronic Resource] 13(10);2018. [↑](#endnote-ref-8)
9. Davies SL, Goodman C, Bunn F, Victor C, Dickinson A, Iliffe S, Gage H, Martin W, Froggatt K. A systematic review of integrated working between care homes and health care services. BMC Health Serv Res. 2011;24(11):320. [↑](#endnote-ref-9)
10. Mason A et al. Integrating funds for health and social care: an evidence review. Journal of health services research & policy 20(3): 177-188;2015. [↑](#endnote-ref-10)
11. Ogbonnaya IN, Keeney AJ. A systematic review of the effectiveness of interagency and cross-system collaborations in the United States to improve child welfare outcomes. Child Youth Serv Rev. 2018;94:225-245. [↑](#endnote-ref-11)
12. Lopez-Carmen V, McCalmana J, Benvenistea T, Askewb D, Spurlingb G, Langhama E, Bainbridgea R. Working together to improve the mental health of indigenous children: A systematic review. Child Youth Serv Rev. 2019;104:104408. [↑](#endnote-ref-12)
13. Whiteford, H., et al. System-level intersectoral linkages between the mental health and non-clinical support sectors: A qualitative systematic review. Australian and New Zealand Journal of Psychiatry 48(10):895-906;2014. [↑](#endnote-ref-13)
14. Martin-Misener R, Valaitis R, Wong ST, Macdonald M, Meagher-Stewart D, Kaczorowski J, O-Mara L, Savage R, Austin P; Strengthening Primary Health Care through Public Health and Primary Care Collaborations Team. A scoping literature review of collaboration between primary care and public health. Prim Health Care Res Dev. 2012 Oct;13(4):327-46. [↑](#endnote-ref-14)
15. Cameron A, Lart R, Bostock L, Coomber C. Factors that promote and hinder joint and integrated working between health and social care services: a review of research literature. Health Soc Care Community. 2014 May;22(3):225-33. [↑](#endnote-ref-15)
16. Winters S, Magalhaes L, Kinsella EA, Kothari A. Cross-sector provision in health and social care: an umbrella review. Int J Integr Care. 2016;16(1):1-19. [↑](#endnote-ref-16)
17. Roussos ST, Fawcett SB. A review of collaborative partnerships as a strategy for improving community health. Annu Rev Public Health. 2000;21:369-402. [↑](#endnote-ref-17)
18. Cooper M, Evens Y, Pybis J. Interagency collaboration in children and young people's mental health: a systematic review of outcomes, facilitating factors and inhibiting factors. Child Care Health Dev. 2016 May;42(3):325-42. [↑](#endnote-ref-18)
19. Sloper, P. Facilitators and barriers for co-ordinated multi-agency services. Child: Care. Health and Development. 2004; 30(6): 571–80. [↑](#endnote-ref-19)
20. Errecaborde KM et al. Factors that enable effective One Health collaborations - A scoping review of the literature. PLoS ONE [Electronic Resource] 14(12);2019. [↑](#endnote-ref-20)
21. Dowling B, Powell M, Glendinning C. Conceptualising successful partnerships. Health and Social Care in the Community. 2004;12(4):309-317. [↑](#endnote-ref-21)
