# Supplementary material for: The impacts of collaboration between local health care and non-health care organizations and factors shaping how they work: a systematic review of reviews
Source: BMC Public Health. 2021 Apr 19;21:753. doi: 10.1186/s12889-021-10630-1 (PMC8054696; doi:10.1186/s12889-021-10630-1)
Supplement: Supplementary file 5 — Additional file 5: Table S4. Summary of evidence on factors influencing collaboration functioning [file 12889_2021_10630_MOESM5_ESM.docx]

**TABLE S4: summary of evidence on factors influencing collaboration functioning**

| **Paper** | **Factors influencing collaboration functioning** | |
| --- | --- | --- |
| *Studies reporting on generic factors* | | |
| Andersson et al (2011) Organizational approaches to collaboration in vocational rehabilitation-an international literature review^[[1]](#endnote-1)^ | - Communication (eg lack of communication can be a barrier) - Trust (eg trust can support collaboration) - ‘Territoriality’ (eg competition between agencies can be a barrier) - Shared aims (eg shared goals for collaboration can support collaboration) - Commitment (eg lack of involvement from key actors can be a barrier) - Rules and regulations (eg different rules on confidentiality can be a barrier) - Leadership (eg leaders who can overcome organizational barriers can support collaboration) | |
| Cameron et al (2014). Factors that promote and hinder joint and integrated working between health and social care services: a review of research literature^[[2]](#endnote-2)^ | *Organizational*   - Aims and objectives (eg shared aims can support collaboration) - Roles and responsibilities (eg lack of understanding of other agencies’ roles can be a barrier) - Flexibility (eg flexibility for staff to work together can support collaboration) - Organisational ‘difference’ (eg conflicting agendas can be a barrier) - Communication and information sharing (eg effective communication can support collaboration) - Co-location (eg co-located teams can support collaboration) - Strong management and ‘appropriate’ professional support (eg different management structures can be a barrier) - History of joint working (eg existing relationships can support collaboration)   *Cultural and professional*   - Conflicting ideologies (eg conflict between medical and social work professions) - Trust and respect (eg lack of trust in other agencies or professions can be a barrier) - Team building (eg joint training between agencies can support collaboration)   *Wider context*   - Organizational change (eg reorganizations can be a barrier) - Financial uncertainty (eg short-term budgets can be a barrier) | |
| Corbin (2016). What makes intersectoral partnerships for health promotion work? A review of the international literature^[[3]](#endnote-3)^ | - Partnership resources (eg time and skills support collaboration) - Mission and purpose (a shared mission can support collaboration) - Financial resources (eg lack of financial resources can be a barrier) - Leadership (eg effective leadership can support collaboration) - Communication (eg quality communication can support collaboration) - Roles and responsibilities (eg role clarity can support collaboration) - Interaction between individual and partnership aims (eg closer alignment can support collaboration) - Partnership tasks (eg implementing tasks to support partnership goals or functioning can support collaboration) - External context (eg lack of political support can be a barrier) - Partnership impact (eg producing results can support collaboration) | |
| Gannon-Leary et al (2006). Collaboration and partnership: A review and reflections on a national project to join up local services in England^[[4]](#endnote-4)^ | - Vision and engagement (eg a clear vision can support collaboration) - Governance (eg boundary conflicts between organizations can be a barrier) - Resources and capacity (eg time and resources can support collaboration) - Relationships (eg interpersonal and interorganizational relationships can support collaboration) | |
| Green et al (2014). Cross-sector collaborations in Aboriginal and Torres Strait Islander childhood disability: A systematic integrative review and theory-based synthesis^[[5]](#endnote-5)^ | *Government level*   - Structure of government agencies (eg fragmentation of departments can be a barrier to collaboration) - Policy collaboration (eg policy frameworks supporting collaboration between sectors can support local collaboration)   *Organizational level*   - Communication and awareness (eg awareness of other agencies can support collaboration) - Role clarity and responsibility (eg lack of role clarity can be a barrier) - Financial and human resources (eg lack of funding can be a barrier) - Service delivery setting (eg culturally sensitive services can support collaboration)   *Service provider level*   - Relationships (eg a linking role between agencies can support collaboration) - Shared professional learning (eg interprofessional training can support collaboration) | |
| Guglielmin et al (2018). A scoping review of the implementation of health in all policies at the local level^[[6]](#endnote-6)^ | - Funding (eg lack of funding can be a barrier) - Shared vision across sectors (eg establishing a shared vision can support collaboration) - National leadership (eg national policy emphasizing health inequalities can support local collaboration to address them) - Ownership and accountability (eg lack of ownership can be a barrier) - Local leadership and dedicated staff (eg lack of dedicated staff can be a barrier) - Health impact assessment (eg implementing health impact assessments can support collaboration) - Use of indicators (eg lack of data for health impact assessments can be a barrier) | |
| Winters et al (2016). Cross-sector provision in health and social care: an umbrella review^[[7]](#endnote-7)^ | - Consumer-centered (eg involving people using services can support collaboration) - Shared vision (eg lack of shared vision can be a barrier) - Leadership (eg effective leadership can support collaboration) - Communication (eg poor communication between partners can be a barrier) - Resources (eg having adequate resources can support collaboration) - History and context (eg history of partnership can support collaboration) - Linkages between sectors (eg shared training and regular meetings can support collaboration) - Role clarity (eg clarifying roles within partnership can support collaboration) | |
| Mackie and Darvill (2016). Factors enabling implementation of integrated health and social care: a systematic review^[[8]](#endnote-8)^ | - Co-location of staff and teamwork (eg co-location can support collaboration) - Communication (eg communication between staff can support collaboration) - Organizational processes (eg fragmentation between organizations can be a barrier) - Management support and leadership (eg leadership support can support collaboration) - Resources and capacity (eg a lack of resources can create additional workload and be a barrier) - National policy (eg national payment systems and incentives can be a barrier) - IT systems (eg lack of shared IT systems can be a barrier) | |
| Martin-Misener et al (2012). Strengthening Primary Health Care through Public Health and Primary Care Collaborations Team. A scoping literature review of collaboration between primary care and public health^[[9]](#endnote-9)^ | *Systemic:*   - Policy context (eg policies mandating partnership working can support collaboration) - Funding and resources (eg lack of resources can be a barrier) - Power and control (eg territorial conflicts can be a barrier) - Education and training (eg shared training can support collaboration)   *Organizational:*   - Common agenda (eg a lack of common agenda can be a barrier) - Knowledge and resources (eg lack of resources can be a barrier) - Leadership, management, and accountability (eg developing inclusive governance committees can support collaboration) - Geographical proximity (eg co-location can support collaboration) - Shared protocols, tools, and information sharing (eg shared information systems can support collaboration)   *Interactional:*   - Shared purpose and philosophy (eg shared values can support collaboration) - Clear roles (eg clarity on roles and can support collaboration) - Positive relationships (eg poor relationships can hinder collaboration) - Effective communication and decision-making (eg open communication and decision-making can support collaboration) | |
| Perkins et al (2010). ‘What counts is what works’? New Labour and partnerships in public health^[[10]](#endnote-10)^ | - Engagement of senior management (eg lack of engagement can be a barrier) - Financial and human resources (eg lack of resources can be a barrier) - Sharing information and best practice (eg information sharing can support collaboration) - Wider context (eg shifting policy context can be a barrier) - Geographical boundaries of agencies (eg lack of shared boundaries can be a barrier) | |
| Rantala et al (2014). Intersectoral action: local governments promoting health^[[11]](#endnote-11)^ | - National or international influences (eg national policy can support local action) - Political context (eg local political will can support collaboration) - Local mechanisms for ISA (eg health impact assessments can support collaboration) - Engagement (eg engagement with non-health sectors and other government agencies is needed to support ISA) - Information sharing (eg sharing information can support collaboration) | |
| Savic et al (2017). Strategies to facilitate integrated care for people with alcohol and other drug problems: a systematic review^[[12]](#endnote-12)^ | - System investment (eg lack of investment in community services can be a barrier) - Government partnerships (eg inter-departmental partnerships can lead to policies, programs, and investments that can support collaboration) - Service contracts (eg government contracts mandating partnership working can support collaboration) - Inter-agency relationships (eg positive relationships can support collaboration) - Shared purpose, values, and priorities (eg shared values between organizations can support collaboration) - Co-location of services (eg co-location can support collaboration, but also can present barriers, such as additional workload) - Staff training (eg staff training in joint working can support collaboration) - Information sharing (eg shared IT systems can support collaboration) - Perceptions of quality in partner agencies (eg lack of confidence in other organizations can be a barrier) - Interprofessional networks (eg territorialism can be a barrier) | |
| *Studies reporting on supportive and/or constraining factors* | | |
| Cooper et al (2016). Interagency collaboration in children and young people's mental health: a systematic review of outcomes, facilitating factors and inhibiting factors^[[13]](#endnote-13)^ | Supportive factors:   - Good communication - Joint training - Good understanding of other sectors and processes - Mutual valuing, respect, and trust - Senior management support - Protocols on interagency collaboration (eg on data sharing) - A named ‘link person’ | Constraining factors:   - Inadequate resources - Poor communication - Lack of valuing, respect, and trust - Differing perspectives or cultures - Poor understanding across professionals and services - Confidentiality issues (eg unable or unwilling to share information) |
| Davies et al (2011). A systematic review of integrated working between care homes and health care services^[[14]](#endnote-14)^ | Supportive factors:   - Health care input and training valued by care homes - ‘Bottom up’ approach to staff training so that all levels of staff are involved - Health care professionals acting as a advocate for care homes - Health care professionals acting as facilitators for sharing good practice and enabling care home staff to network - Health care professionals promoting better access to services for the care home - Care home managers supporting staff access to training | Constraining factors:   - Lack of trust and confidence between health care and care home staff - Lack of access to health care services - High staff turnover and lack of access to training - Lack of staff knowledge and confidence - Care homes being professionally isolated - Lack of teamwork in care homes |
| Herdiana et al (2018). Intersectoral collaboration for the prevention and control of vector borne diseases to support the implementation of a global strategy: a systematic review^[[15]](#endnote-15)^ | Supportive factors:   - Shared vision (eg agreement on outcomes) - Management (eg implementation capacity) - Relationships (eg consistent communication) - Approach (eg using a participatory approach) - Resources (eg adequate financial and technical support) | Constraining factors:   - Political differences - Poor communication and coordination - Financial constraints - Lack of local commitment Insufficient or irregular supplies - Lack of tangible benefits - Weak monitoring or evaluation - Different geographical areas - Professional attitudes and behaviors - Inaccessible area - Poor leadership - Difficulties sharing decision-making and power - Different organizational cultures and histories - Organizational rigidities - Contested planning priorities |
| Seaton et al (2018). Factors that impact the success of interorganizational health promotion collaborations: a scoping review^[[16]](#endnote-16)^ | Supportive factors:   - Shared vision and goals - Leadership (including mechanisms for partners to participate in decision-making) - Member skills and characteristics - Organizational commitment - Resources and technical support - Clear roles and responsibilities - Trust, communication, and relationships - Community engagement | Constraining factors:   - Absence of supportive factors - Government mandates or policy directives to collaborate - Power imbalances between partners |
| Sloper, P (2004). Facilitators and barriers for co-ordinated multi-agency services^[[17]](#endnote-17)^ | Supportive factors: *Service planning:*   - Clear and realistic aims - Clearly defined roles and responsibilities - Commitment of leaders and staff - Strong leadership and multi-agency management structures - Agreed timetable for implementation and incremental approach to change - Linking projects to other planning and decision-making processes - Good communication   *Service implementation and management:*   - Shared and adequate resources - Staff with the right experience and approach - Joint training and team building - Appropriate support and supervision of staff - Service monitoring and evaluation   *Other:*   - Cultural factors (eg understanding partners’ aims and functions) - Wider context (eg history of partnership working can support collaboration) | Constraining factors:   - Lack of clarity on roles and responsibilities - Differences in organizational aims - Lack of consensus on aims or overambitious aims - Lack of commitment and support from senior managers |
| Whiteford et al (2014). System-level intersectoral linkages between the mental health and non-clinical support sectors: A qualitative systematic review^[[18]](#endnote-18)^ | Supportive factors:   - Communication between sectors - Strong leadership (eg mechanisms for resolving conflicts) - Shared perspective or mutual understanding - Co-location and service linkages - Overarching plan and coordination (eg a coordinating body between organizations) - Monitoring (eg service evaluation) - Engagement - Competitive grants (eg can garner interest in participating in collaboration activities or reforms) | Constraining factors:   - Lack of funding and resources - Differences in perspective or lack of clarity on roles - Barriers to information sharing - Inappropriate referrals (eg on sector fearing increased activity) |
| Wildridge et al (2004). How to create successful partnerships: a review of the literature^[[19]](#endnote-19)^ | Supportive factors:   - Shared vision - Trust - Communication - Effective decision-making and accountability - Effective change management - Legislation (eg that creates flexible rules for organizations to collaborate) - Supportive environment (eg history of collaboration) - Membership characteristics (eg appropriate members involved) - Supportive processes and structures (eg clear roles and guidelines) - Sufficient resources | Constraining factors:   - Lack of motivation or perverse incentives - Insufficient resources - Power imbalances - Resource conflicts and ‘cost shifting’ - Cultural issues (eg between staff in health and social care organizations) - Structural differences - Accountability and decision-making differences |
| Bagnall et al (2019). Whole systems approaches to obesity and other complex public health challenges: a systematic review^[[20]](#endnote-20)^ | Supportive factors:   - Strong leadership - Community engagement (eg to identify health needs and potential solutions) - Relationships and trust - Community capacity - Good governance and shared values - A effective collaborative team (eg early participation of key stakeholders) - Consistency in language across organizations (eg to overcome differences in values and stuctures) - Embedding initiatives in broader policy context - Local evaluations (eg to inform interventions) - Sufficient financial support and resources | NA |
| Dowling et al (2004). Conceptualising successful partnerships^[[21]](#endnote-21)^ | Supportive factors:   - Engagement of partners - Agreement on need for and aims of partnership - Trust and respect among partners - Satisfactory accountability arrangements - Adequate leadership and management - Wider context (eg financial climate and legal and institutional structures shape partnership success) | NA |
| Errecaborde et al (2019). Factors that enable effective one health collaborations: a scoping review of the literature^[[22]](#endnote-22)^ | Supportive factors:  *Individual level*   - Education and training - ‘Just in time’ training - Existing experience and relationships   *Organizational level*   - Structures and policies (eg shared response guidelines) - Systems (eg shared information systems) - Culture (eg engaged leadership) - Human resources (eg staff with defined roles and responsibilities)   *Network level*   - Network structures (eg coordination mechanisms between organizations) - Network relationships (eg defined roles and responsibilities between partners) - Resources (eg financial and human resources) - Political environment (eg political will) - Network leadership (eg shared decision-making) - Network management (eg established lines of communication between organizations) - Monitoring and evaluation - Resource mobilization and allocation (eg financial and human resources) | NA |
| Foster-Fishman et al (2001). Building collaborative capacity in community coalitions: a review and integrative framework^[[23]](#endnote-23)^ | Supportive factors:  *Member capacity*   - Works collaboratively with others - Ability to build effective programs - Ability to build an effective coalition infrastructure - Holds positive attitudes about collaboration - Committed to target issues - Holds positive attitudes about other stakeholders - Holds positive attitudes about self (eg as a legitimate partner) - Access to member capacity - Coalition supports member involvement - Coalition builds member capacity (eg provides technical support)   *Relational capacity*   - Develops a positive working climate - Develops a shared vision - Promotes power sharing - Values diversity (eg individual and group differences appreciated) - Develops positive external relationships   *Organizational capacity*   - Effective leadership (eg skilled at conflict resolution) - Task-oriented work environment - Formalized procedures (eg clear member roles and responsibilities) - Effective communication (eg timely information sharing) - Sufficient resources - Continuous improvement orientation   *Programmatic capacity*   - Clear objectives - Realistic goals - Driven by and addresses community needs | NA |
| Lopez-Carmen et al (2019) Working together to improve the mental health of indigenous children: A systematic review^[[24]](#endnote-24)^ | Supportive factors:   - Community involvement - Resources and access (eg increased organizational funding for integrated interventions) - Collaboration between services and systems (eg sharing information between sectors) - Strong relationships - Cultural sensitivity (eg knowledge of historical contexts and trauma of indigenous populations) - Organizational and staff capacity (eg funding and resources for teams to collaborate) | NA |
| Zakocs and Edwards (2006). What explains community coalition effectiveness? A review of the literature^[[25]](#endnote-25)^ | Supportive factors:   - Formalization/rules - Leadership style - Active member participation - Diverse membership - Member agency collaboration - Group cohesion - Open/frequent communication channels - Intensity/scope of actions implemented - Task/goal focused climate - Staff time devoted to tasks - Conflict management - Agency member types - Participatory decision-making - Member experience/expertise - Member benefits - Training/technical assistance - Sectors (agencies) represented - Member ownership/commitment - Effective administration - Efficient use of resources - Target small geographic areas - Coalition readiness - Collaboration before coalition - Comprehensive vision - Supportive organizational climate - Trust - Recognize life cycles - Establish priorities - Innovation - Researcher driven - Written assessment/implement plan - Data-driven planning - Gained political support - Prevention focused - Used media to promote coalition - Used environmental strategies - Dedicated project director - Lead agency known entity - Lead agency noncompetitor - Lead agency director supportive - Length of time members involved - Membership size - Member-perceived fairness - Member satisfaction - Member empowerment - Member sense of community - Member perceived community problems - Member anger/aggression - Member self-discovery - Member independence - Member knowledge of other agencies - Staff relationships with members - Staff expertise/experience - Paid coordinator - Personnel barriers | NA |
| Roussos and Fawcett (2000). A review of collaborative partnerships as a strategy for improving community health^[[26]](#endnote-26)^ | Supportive factors:   - Clear vision and mission - Action planning for community and systems change - Developing and supporting leadership - Measuring progress, including on intermediate outcomes - Technical assistance (eg training in community health assessments or evaluation) - Financial resources - Making outcomes matter (eg by promoting partnership outcomes to community members and others) | NA |
|  | Contextual factors:   - Social and economic factors - Community social capital - Partnership context (eg history of collaboration, time for partnership) - Community control in agenda setting | |
| Auschra C (2018). Barriers to the integration of care in inter-organisational settings:  a literature review^[[27]](#endnote-27)^ | NA | Constraining factors:  *Regulation and administration*   - Regulatory issues (eg data sharing) - Historical context (eg lack of history of collaboration) - Administrative boundaries   *Funding*   - Insufficient funding - Fear of cost shifting   *Inter-organizational*   - Lack of leadership coordination - Organizational differences (eg in decision-making processes) - Power imbalances - Conflicting aims - Failure to include key partners   *Organizational*   - Organizational agenda differs from collective interests - Cultural differences - Previous experiences between organizations   *Service delivery*   - Professional differences - Poor communication - Lack of trust - Lack of mutual understanding - Resistance to change - Lack of technical standards (eg for data sharing)   *Clinical*   - Lack of information sharing - Confidentiality issues (eg leading to lack of information sharing) |
| Mason et al (2015). Integrating funds for health and social care: an evidence review^[[28]](#endnote-28)^ | NA | Constraining factors:   - Challenges breaking down service barriers (eg professional opposition) - Relational issues (eg poor relationships between sectors) - Difficulty engaging service users - Information technology issues (eg incompatible systems) - Accountability and structural differences (eg challenges transferring funds between organizations) |
| Williams I (2009). Offender health and social care: a review of the evidence on inter-agency collaboration^[[29]](#endnote-29)^ | NA | Constraining factors:   - Structural incompatibility (eg health and social care reluctance to work with criminal justice agencies) - Procedural differences (eg different approaches to engaging offenders) - Different professional values (eg between health and criminal justice) - Information sharing (eg difficulties sharing information, both technical and related to professional differences) |

1. Andersson J, Ahgren B, Axelsson SB, Eriksson A, Axelsson R. Organizational approaches to collaboration in vocational rehabilitation-an international literature review. Int J Integr Care. 2011 Oct;11:e137. [↑](#endnote-ref-1)
2. Cameron A, Lart R, Bostock L, Coomber C. Factors that promote and hinder joint and integrated working between health and social care services: a review of research literature. Health Soc Care Community. 2014 May;22(3):225-33. [↑](#endnote-ref-2)
3. Corbin JH, Jones J, Barry MM. What makes intersectoral partnerships for health promotion work? A review of the international literature. Health Promotion International. 2016;33(1):4-26 [↑](#endnote-ref-3)
4. Gannon-Leary P, Baines S, Wilson R. Collaboration and partnership: A review and reflections on a national project to join up local services in England. Journal of Interprofessional Care. 2006;20(6):665-674. [↑](#endnote-ref-4)
5. Green A et al. Cross-sector collaborations in Aboriginal and Torres Strait Islander childhood disability: A systematic integrative review and theory-based synthesis. International Journal for Equity in Health 13(1);2014. [↑](#endnote-ref-5)
6. Guglielmin, M., et al. A scoping review of the implementation of health in all policies at the local level. Health Policy 122(3): 284-292;2018. [↑](#endnote-ref-6)
7. Winters S, Magalhaes L, Kinsella EA, Kothari A. Cross-sector provision in health and social care: an umbrella review. Int J Integr Care. 2016;16(1):1-19. [↑](#endnote-ref-7)
8. Mackie S, Darvill A. Factors enabling implementation of integrated health and social care: a systematic review. British Journal of Community Nursing. 2016;21(2):82-87. [↑](#endnote-ref-8)
9. Martin-Misener R, Valaitis R, Wong ST, Macdonald M, Meagher-Stewart D, Kaczorowski J, O-Mara L, Savage R, Austin P; Strengthening Primary Health Care through Public Health and Primary Care Collaborations Team. A scoping literature review of collaboration between primary care and public health. Prim Health Care Res Dev. 2012 Oct;13(4):327-46. [↑](#endnote-ref-9)
10. Perkins N, Smith K, Hunter DJ, Bambra C, Joyce K. ‘What counts is what works’? New Labour and partnerships in public health. Politics and Policy, 2010;38(1):101-117. [↑](#endnote-ref-10)
11. Rantala R, Bortz M, Armada F. Intersectoral action: local governments promoting health. Health Promotion International. 2014;29(Suppl 1):i92i102. [↑](#endnote-ref-11)
12. Savic M, Best D, Manning V, Lubman D. Strategies to facilitate integrated care for people with alcohol and other drug problems: a systematic review. Subst Abuse Treat Prev Policy. 2017;12(1):19. [↑](#endnote-ref-12)
13. Cooper M, Evens Y, Pybis J. Interagency collaboration in children and young people's mental health: a systematic review of outcomes, facilitating factors and inhibiting factors. Child Care Health Dev. 2016 May;42(3):325-42. [↑](#endnote-ref-13)
14. Davies SL, Goodman C, Bunn F, Victor C, Dickinson A, Iliffe S, Gage H, Martin W, Froggatt K. A systematic review of integrated working between care homes and health care services. BMC Health Serv Res. 2011;24(11):320. [↑](#endnote-ref-14)
15. Herdiana, H., et al. Intersectoral collaboration for the prevention and control of vector borne diseases to support the implementation of a global strategy: A systematic review. PLoS ONE [Electronic Resource] 13(10);2018. [↑](#endnote-ref-15)
16. Seaton CL, Holm N, Bottorff JL, Jones-Bricker M, Errey S, Caperchione CM, Lamont S, Johnson ST, Healy T. Factors that impact the success of interorganizational health promotion collaborations: a scoping review. Am J Health Promot. 2018 May;32(4):1095-1109. [↑](#endnote-ref-16)
17. Sloper, P. Facilitators and barriers for co-ordinated multi-agency services. Child: Care. Health and Development. 2004; 30(6): 571–80. [↑](#endnote-ref-17)
18. Whiteford, H., et al. System-level intersectoral linkages between the mental health and non-clinical support sectors: A qualitative systematic review. Australian and New Zealand Journal of Psychiatry 48(10):895-906;2014. [↑](#endnote-ref-18)
19. Wildridge V, Childs S, Cawthra L, Madge B. How to create successful partnerships: a review of the literature. Health Information and Libraries Journal. 2004;21:3–19. [↑](#endnote-ref-19)
20. Bagnall AM, Radley D, Jones R, Gately P, Nobles J, Van Dijk M, Blackshaw J, Montel S, Sahota P. Whole systems approaches to obesity and other complex public health challenges: a systematic review. BMC Public Health. 2019;19(1):8. [↑](#endnote-ref-20)
21. Dowling B, Powell M, Glendinning C. Conceptualising successful partnerships. Health and Social Care in the Community. 2004;12(4):309-317. [↑](#endnote-ref-21)
22. Errecaborde KM et al. Factors that enable effective One Health collaborations - A scoping review of the literature. PLoS ONE [Electronic Resource] 14(12);2019. [↑](#endnote-ref-22)
23. Foster-Fishman PG, Berkowitz SL, Lounsbury DW, Jacobson S, Allen NA. Building collaborative capacity in community coalitions: a review and integrative framework. Am J Community Psychol. 2001;29(2):241-61. [↑](#endnote-ref-23)
24. Lopez-Carmen V, McCalmana J, Benvenistea T, Askewb D, Spurlingb G, Langhama E, Bainbridgea R. Working together to improve the mental health of indigenous children: A systematic review. Child Youth Serv Rev. 2019;104:104408. [↑](#endnote-ref-24)
25. Zakocs RC, Edwards EM. What explains community coalition effectiveness? A review of the literature. Am J Prev Med. 30(4):351-61;2006. [↑](#endnote-ref-25)
26. Roussos ST, Fawcett SB. A review of collaborative partnerships as a strategy for improving community health. Annu Rev Public Health. 2000;21:369-402. [↑](#endnote-ref-26)
27. Auschra C. Barriers to the integration of care in inter-organisational settings: a literature review. International Journal of Integrated Care, 2018;18(1)5:1–14. [↑](#endnote-ref-27)
28. Mason A et al. Integrating funds for health and social care: an evidence review. Journal of health services research & policy 20(3): 177-188;2015. [↑](#endnote-ref-28)
29. Williams I. Offender health and social care: a review of the evidence on inter-agency collaboration. Health and Social Care in the Community. 2009;17(6):573–580. [↑](#endnote-ref-29)
